# The oncogenic circular RNA *circ_63706* is a potential therapeutic target in sonic hedgehog-subtype childhood medulloblastomas

**DOI:** 10.1186/s40478-023-01521-0

**Published:** 2023-03-10

**Authors:** Keisuke Katsushima, Rudramani Pokhrel, Iqbal Mahmud, Menglang Yuan, Rabi Murad, Prabin Baral, Rui Zhou, Prem Chapagain, Timothy Garrett, Stacie Stapleton, George Jallo, Chetan Bettegowda, Eric Raabe, Robert J. Wechsler-Reya, Charles G. Eberhart, Ranjan J. Perera

**Affiliations:** 1 Department of Oncology, Sidney Kimmel Comprehensive Cancer Center, School of Medicine, Johns Hopkins University, 1650 Orleans St., Baltimore, MD 21231 USA; 2 Johns Hopkins All Children’s Hospital, St. Petersburg, USA; 3 Department Pathology, Immunology and Laboratory Medicine, College of Medicine, University of Florida, Gainesville, USA; 4 Present Address: Department of Bioinformatics and Computational Biology, University of Texas MD Anderson Cancer Center, Houston, USA; 5 Sanford Burnham Prebys Medical Discovery Institute, La Jolla, USA; 6 Department of Physics, Florida International University, Miami, USA; 7 Biomolecular Sciences Institute, Florida International University, Miami, USA; 8 Department of Neurosurgery, Johns Hopkins University School of Medicine, Baltimore, USA; 9 Department of Pathology, Johns Hopkins University School of Medicine, Baltimore, USA; 10 Herbert Irving Comprehensive Cancer Center, Columbia University Medical, New York, USA

**Keywords:** Medulloblastoma, Circular RNA, Sonic hedgehog, Global lipidome

## Abstract

Medulloblastoma (MB) develops through various genetic, epigenetic, and non-coding (nc) RNA-related mechanisms, but the roles played by ncRNAs, particularly circular RNAs (circRNAs), remain poorly defined. CircRNAs are increasingly recognized as stable non-coding RNA therapeutic targets in many cancers, but little is known about their function in MBs. To determine medulloblastoma subgroup-specific circRNAs, publicly available RNA sequencing (RNA-seq) data from 175 MB patients were interrogated to identify circRNAs that differentiate between MB subgroups. *circ_63706* was identified as sonic hedgehog (SHH) group-specific, with its expression confirmed by RNA-FISH analysis in clinical tissue samples. The oncogenic function of *circ_63706* was characterized in vitro and in vivo. Further, *circ_63706*-depleted cells were subjected to RNA-seq and lipid profiling to identify its molecular function. Finally, we mapped the *circ_63706* secondary structure using an advanced random forest classification model and modeled a 3D structure to identify its interacting miRNA partner molecules. *Circ_63706* regulates independently of the host coding gene pericentrin (*PCNT*), and its expression is specific to the SHH subgroup. *circ_63706*-deleted cells implanted into mice produced smaller tumors, and mice lived longer than parental cell implants. At the molecular level, *circ_63706*-deleted cells elevated total ceramide and oxidized lipids and reduced total triglyceride. Our study implicates a novel oncogenic circular RNA in the SHH medulloblastoma subgroup and establishes its molecular function and potential as a future therapeutic target.

**Supplementary Information:**

The online version contains supplementary material available at 10.1186/s40478-023-01521-0.

## Introduction

Medulloblastoma (MB) is a highly malignant childhood brain tumor accounting for ~ 20% of all pediatric brain tumors and 63% of intracranial embryonic tumors [8]. Approximately 500 patients are diagnosed with medulloblastoma in the United States each year, of whom 60% are children under fifteen [8]. Advances in next‐generation sequencing and genome‐wide association analyses have unraveled significant heterogeneity in medulloblastoma [12], such that the World Health Organization Classification of Tumors of the Nervous System has for some time classified MBs into molecular subgroups: wingless (WNT)-activated, sonic hedgehog (SHH)-activated and *TP53* wildtype, SHH-activated and *TP53* mutant, and non-WNT/non-SHH [24]. Many studies have discovered reliable molecular markers for these subgroups. However, their degree of overlap, underlying genetics and biology, and intrinsic diversity have yet to be fully identified [29, 45], despite a need to define individual tumors for targeted therapy. There is a compelling clinical need for novel molecular markers and therapeutic targets for specific molecular subgroups to improve outcomes.

Recent studies have identified several medulloblastoma subgroup-specific biomarkers and molecular targets including oncogenes and tumor suppressor genes such as *MYCN, MYC, TP53, CDK6, ALK, GLI1, SNCAIP, OTX2*, and *SNCA *[19]. Fully defining medulloblastoma heterogeneity requires an approach that goes beyond characterizing individual genes, since cancer development represents the product of complex interactions in and between signaling networks and their regulation. Noncoding (nc) RNAs—which represent most of the transcribed genome—may be useful for sub-stratifying MBs. We recently identified significant heterogeneity in long non-coding RNAs (lncRNAs) in MBs by molecular subgroup [11], with *lnc-HLX-2-7* oncogenic in Group 3 (G3) MBs [13] and *Sprightly* in Group 4 (G4) MBs [16].

Circular RNAs (circRNAs) have recently emerged as a class of endogenous tissue- and developmental stage-specific ncRNAs [15]. CircRNAs are exceptionally stable and generally cytoplasmic [15, 33]. CircRNAs are now established as pathogenic in various cancers and have potential as diagnostic or therapeutic targets. In addition, circRNAs are abundant in the mammalian brain [33], so they may be perfect candidates for biomarkers in medulloblastoma. CircRNAs are generated from pre-messenger RNA via back-splicing, where the 3′ and 5′ ends are connected via a covalent bond to form a loop [7] structure devoid of a 5′ cap and 3′ poly(A) tail. Therefore, circRNAs are resistant to degradation by RNases and are abundant in mammalian cells and body fluids [28], providing opportunities for non-invasive sampling for biomarker analysis. CircRNAs can regulate gene expression and translation by sponging RNA-binding proteins and microRNAs (miRNA, miR) [31, 42] and, in some cases, generating a protein through translation [36]. CircRNAs are dysregulated in various cancers, where they mediate cellular proliferation, migration, and invasion. CircRNAs have also been documented in medulloblastoma: two circRNAs (*circ‐SKA3* and *circ‐DTL*) promoted the proliferation, migration, and invasion of medulloblastoma cells in vitro by regulating gene expression [25]. A recent study investigated the oncogenic characteristics of *circ-SKA3*, which increased ID3 expression by decoying miR-326 to promote medulloblastomagenesis [43]. One computational analysis proposed medulloblastoma subgroup-specific circRNAs, but these have yet to be validated experimentally [32]. Further detailed analysis of the circRNA content in medulloblastoma and their subgroup-specific distribution is urgently required to pave the way for new clinical diagnostics and therapeutics. In addition to regulating cancer hallmarks such as proliferation and invasion, several circRNAs have also been shown to modulate lipid synthesis and other metabolism pathways in cancer by altering various miRNAs [41].

Elevated lipid synthesis is a cancer hallmark, since cancer cells require fatty acids, glycerolipids, glycerophospholipids, and cholesterol esters for cellular membrane maintenance and cellular proliferation. For instance, *circ_0057558* expression has been shown to be positively associated with triglyceride (TG) levels in prostate cancer [38] and, using bioinformatics approaches, three circRNA interaction axes were predicted in prostate cancer with unclear roles in cancer metabolism [37]. CircRNAs have also been shown to interact with c-myc [40] and HIF1-α [21] in several cancer models, suggesting a potential role for circRNAs in cancer metabolism. However, the full and probably varied roles of circRNAs in medulloblastoma and metabolism have yet to be characterized.

Here we identified medulloblastoma subgroup-specific circRNAs in 126 MBs through RNA-seq data analysis using the CIRI2 [6] detection pipeline. We combined a biostatistical approach and random forest classification to identify subgroup-specific circRNAs with diagnostic potential. Candidate circRNAs were validated by RT-PCR in cell lines and patient samples. We further tested the SHH subgroup-specificity of one circRNA (*circ_63706*; hsa-PCNT_0003, CircAtlas 2.0) using RNA fluorescence in situ hybridization (FISH) in clinical samples, paving the way for using *circ_63706* as an SHH-specific biomarker. Detailed molecular analysis suggested that *circ_63706* may reprogram global lipid metabolism in MB cells to enhance tumorigenesis. Based on our results, we postulate that oncogenic circRNA *circ_63706* is an important therapeutic target and biomarker for SHH MBs.

## Materials and methods

### RNA sequencing datasets

FASTQ files for RNA-seq data were collected from the European Genome-Phenome Archive (http://www.ebi.ac.uk/ega/, accession number: EGAD00001003279) after obtaining permission from the ICGC Data Access Compliance Office. The data represented 175 medulloblastoma samples [n = 18 WNT, n = 46 SHH, n = 45 Group 3 (G3), and n = 66 Group 4 (G4)].

Later, we used to confirm our initial analysis in a separate dataset (n = 22 WNT, n = 43 SHH, n = 9 G3 and n = 23 G4) obtained from the St. Jude Hospital. We also isolated RNA samples from patient-derived xenografts (PDXs). The Wechsler-Reya lab established the DMB006, DMB012, RCMB28, RCMB32, RCMB38, RCMB40, RCMB45, and RCMB51 PDXs. The Olson lab at the Fred Hutchinson Cancer Research Center established MED211FH, MED511FH, and MED1712FH PDXs. The Milde lab at the German Cancer Research Center (DKFZ) established the BT-084 PDX, and the Cho lab at Oregon Health and Sciences University established the MB002 PDX. The Wechsler-Reya lab maintained all PDXs.

### RNA fluorescence in situ hybridization (RNA-FISH)

RNA was visualized in formalin-fixed, paraffin-embedded tissue (FFPE) sections using the QuantiGene ViewRNA ISH Tissue Assay Kit (Thermo Fisher Scientific, Waltham, MA). Tissue sections were rehydrated and incubated with proteinase K. Subsequently, we incubated the sections with ViewRNA probesets designed to target human *circ_63706* (Thermo Fisher Scientific). Hybridization was performed according to the manufacturer’s instructions.

### siRNA-mediated knockdown

siRNAs targeting *circ_63706* were purchased from Integrated DNA Technologies (Coralville, IA). Cells were transfected with 20 nM siRNA targeting each gene or control non-targeting siRNA (negative control siRNA) (AM4611, Applied Biosystems, Foster City, CA) for 48 h using Lipofectamine RNAiMAX (Thermo Fisher Scientific). Knockdown efficiency was assessed using qRT-PCR. The following siRNAs sequences targeted *circ_63706* (#1): CAGCTGGAGACCCTGAAGGAA and (#2): ACAGCTGGAGACCCTGAAGGA.

### Medulloblastoma xenografts

All mouse studies were performed following the policies and regulations of the Animal Care and Use Committee of Johns Hopkins University, which approved the studies. We established intracranial medulloblastoma xenografts by injecting DAOY, ONS76, and DAOY cells with *circ_63706* knockdown into the cerebellums of NOD-SCID mice (Jackson Laboratory, Bar Harbor, ME). Cerebellar coordinates were − 2 mm from lambda, + 1 mm laterally, and 1.5 mm deep. We evaluated tumor growth with weekly bioluminescence imaging using an in vivo spectral imaging system (IVIS Lumina II, Xenogen, Alameda, CA).

## Results

### Differential expression of circRNAs in medulloblastoma subgroups

**Fig. 1:**
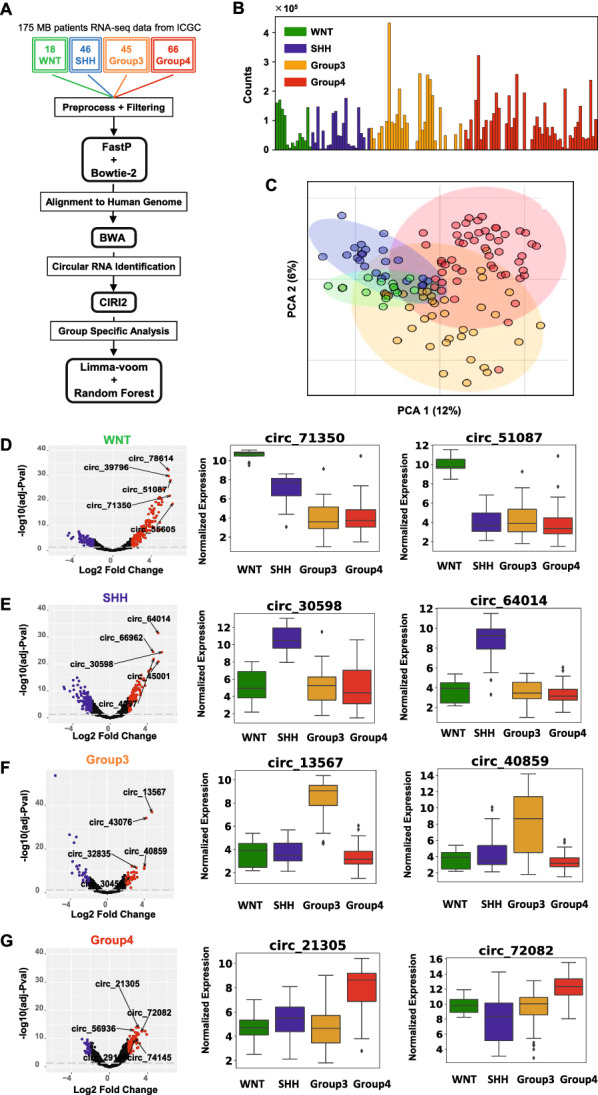
Differential expression of circRNAs in medulloblastoma subgroups. **A** Circular RNA identification pipeline for the 126 medulloblastoma patients in four subgroups. **B** Raw total circular RNA counts across all 126 medulloblastoma patient samples. **C** Principal component clustering of 8925 highly expressed circRNAs. **D**–**G** The red and blue points and shades show significant circular RNAs for a group versus others with an adjusted *p* value < 0.05 and |log2 fold change|> 2. The box plot shows normalized expression for two significant upregulated circRNAs in WNT (**D**), SHH (**E**), G3 (**F**), and G4 (**G**) across all 126 medulloblastoma patients

The circRNA detection pipeline is depicted in Fig. 1A. The pipeline detected 79,099 circRNAs in 175 medulloblastoma samples. After filtering out low count samples, the count matrix contained 8925 circRNAs across 126 samples (n = 14 WNT, n = 23 SHH, n = 37 G3, and n = 52 G4) (Fig. 1B). Two-dimensional principal component analysis (PCA; Fig. 1C) showed group-specific sample clustering, with all sample groups tending to overlap (95% CIs, marked by shaded areas). Clustering of differentially expressed circRNAs (Additional file 3: Figs. S1–S8) mirrored the PCA findings.

Nevertheless, several circRNAs were differentially expressed between subgroups (|log2-fold change|> 2 and FDR < 0.05; Fig. 1D–G). Since we sought to identify highly statistically significant group-specific circRNAs, we focused on differentially expressed (upregulated) circRNAs in a given group *vs.* the other three groups. 114 circRNAs in the WNT subgroup, 48 in the SHH group, 13 in G3, and 21 in G4 MBs were upregulated (Additional file 3: Fig. S2B). Figure 1D–G illustrate the top two differentially expressed circRNAs in each subgroup identified by the *limma-voom* method (Additional file 1).

Since only 13 circRNAs were significantly upregulated in G3 MBs (log2-fold change > 2 and FDR < 0.05), we took a similar number (n = 15) of upregulated circRNAs from other groups for experimental validation and functional studies (i.e., 58 significantly upregulated circRNAs, Additional file 4: Table S2). Data were ordered according to decreasing log2-fold change values to select the top 15 circRNAs. These 58 circRNAs separated the 126 medulloblastoma samples according to medulloblastoma subgroup, especially SHH and WNT from G3 and G4 tumors (Additional file 3: Fig. S3; expression in Additional file 3: Figs. S4–S7).

### Subgroup-specific marker genes from random forest (RF) classification

The random forest (RF) machine learning algorithm provides efficient and high predictive accuracy for many data types, including clinical and molecular data. RF is particularly useful for genomic data analysis, which is typically of small sample size but high feature dimension. Differential expression (DE) analysis packages are not optimized for circular RNA analysis due to inherent complications with normalization. Therefore, to validate the top 58 circRNAs obtained from DE analysis, we built and applied an RF model to identify subgroup-specific circRNAs. The model used 460 circRNAs across 126 samples, selected using recursive feature elimination (RFE) with RF. The heatmap of loading coefficient of the top 28 circRNAs contributing to the classification model appears in Additional file 3: Fig. S8A. By evaluating the contrast of loading coefficients and expression in normalized data (Additional file 3: Fig. S9), 16 subgroup-specific circRNAs were finally identified (n = 5 in WNT, n = 5 in SHH, n = 3 in G3, and n = 3 in G4; Additional file 3: Fig. S8B), nine of which (in bold and italicized letter) were also present in the top 58 differentially expressed genes. The area under the receiver operating characteristics (AUC-ROC) curve was > 95%, suggesting a high predictive accuracy for the RF classification model (Additional file 3: Fig. S8C).

### Validation of circRNAs by quantitative RT-PCR

The two combined analytic methods identified 65 subgroup-specific circRNAs (Additional file 4: Table S2), which were subsequently filtered to a final 12 circRNAs with higher abundance in raw count data (n = 4 SHH, n = 5 G3, and n = 3 G4; Additional file 4: Table S3). We designed primer pairs to cover the circRNA junction sequence using NCBI Primer-BLAST and Primer3Plus tools (Additional file 4: Table S1), and *ACTB* was used as a control.

**Fig. 2:**
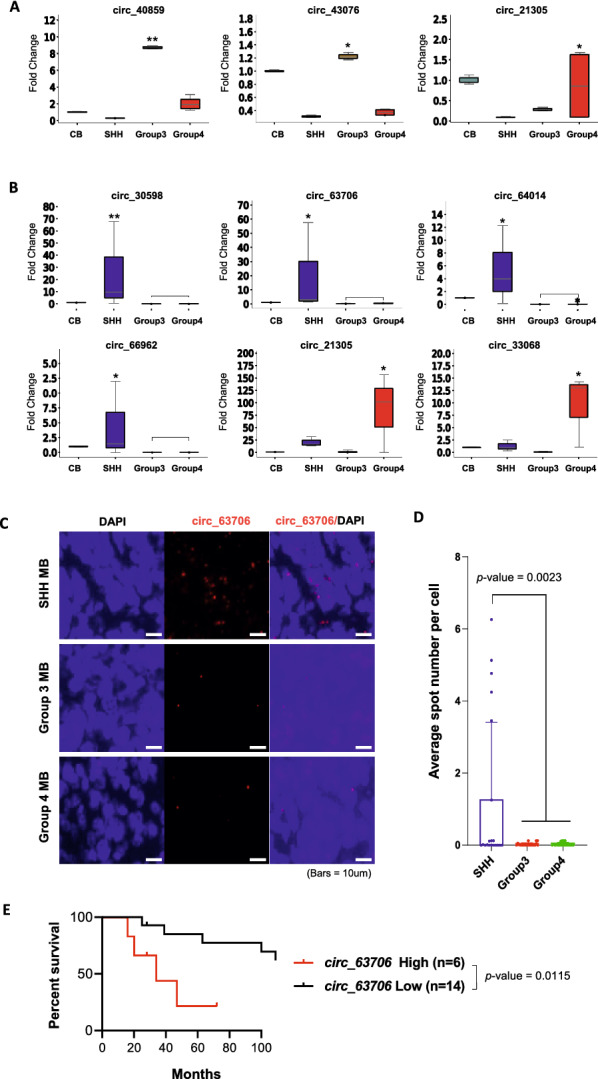
CircRNAs validated by qRT-PCR and RNA-FISH in cell lines, PDXs, and clinical MB patient samples. **A** and **B** Fold change for normal cerebellum (CB) in medulloblastoma cell line samples (**A**) and PDX samples (**B**). Values indicate fold change relative to cerebellum. *p < 0.05, **p < 0.01, Kruskal–Wallis analysis. **C** and **D** RNA-FISH confirms that *circ_63706* expression is specific to SHH medulloblastoma patients. **C** Representative RNA-FISH analysis of *circ_63706* in medulloblastoma tissues. RNA-FISH analysis of *circ_63706* in SHH medulloblastoma patients (top panels) and G3 and G4 medulloblastoma patients (lower panels). **D** Spot numbers relate to *circ_63706* per cell in SHH, G3, and G4 medulloblastoma patients. n = 20, *p < 0.01, Student’s *t*-test. **E** Kaplan–Meier survival curves of SHH medulloblastoma patients according to *circ_63706* expression. SHH medulloblastoma patient samples were divided into *circ_63706* high (average spot number per cell > 1.0) and *circ_63706* low (average spot number per cell < 1.0)

The Ct values for the expression of all circRNAs across all cell lines and PDX samples are provided in Additional file 4: Table S4. Of the 12 circRNAs, only three had subgroup-specific overexpression when validated in cell lines (Fig. 2A); two in G3 (*circ_40859*, *circ_43076*) and one (*circ_21305*) in G4. Six circRNAs were validated in PDX samples, including all four SHH-specific circRNAs (*circ_30598*, *circ_63706, circ_64014*, and *circ_66962*) and two G4-specific circRNAs (*circ_21305* and *circ_33068*) (Fig. 2B).

We predicted the protein-coding potential of these six circRNAs using the RNAsamba tool [3], which uses a neural network classification model. The output of this model is summarized in Additional file 4: Table S5. Out of all six circRNAs, five had protein coding potential (circRNAs *circ_33068* and *circ_63706* had > 90% coding potential) and *circ_*30598 had no coding potential.

### *Circ_63706* expression is specific to SHH MBs

Of the six SHH subgroup-specific circRNAs (*circ_30598*, *circ_63706, circ_64014*, *circ_66962, circ_21305*, and *circ_33068*), only *circ_63706* showed statistically significantly higher expression in the SHH subgroup by qRT-PCR in PDX samples compared with the other three groups. The cell line results are shown in Additional file 3: Fig. S10. Therefore, we decided to focus on *circ_63706* and further confirmed its expression by RNA-FISH in formalin-fixed paraffin-embedded tissue samples from patients with MBs. Out of 20 medulloblastoma samples, *circ_63706* was highly expressed in six SHH samples but not in any G3 or G4 MBs (Fig. 2C). Quantitative analysis of the tissues further confirmed significantly higher *circ_63706* expression in SHH MBs than in G3 and G4 MBs, with high specificity (100%; *p* < 0.0023; Fig. 2D). Importantly, the significantly higher expression level of *circ_63706* in SHH MBs was further confirmed in an independent sample set using the St. Jude Cloud [27] (Additional file 3: Fig. S11). Survival analysis using clinical data reported in our previous study showed that *circ_63706* overexpression was associated with poor patient outcomes in SHH MB (Fig. 2E). Collectively, our analyses suggest that *circ_63706* expression is specific to SHH MBs and can be detected using an assay readily applicable to the clinical setting (FISH).

### Functional characterization of *circ_63706* in SHH cell lines and PDXs

**Fig. 3:**
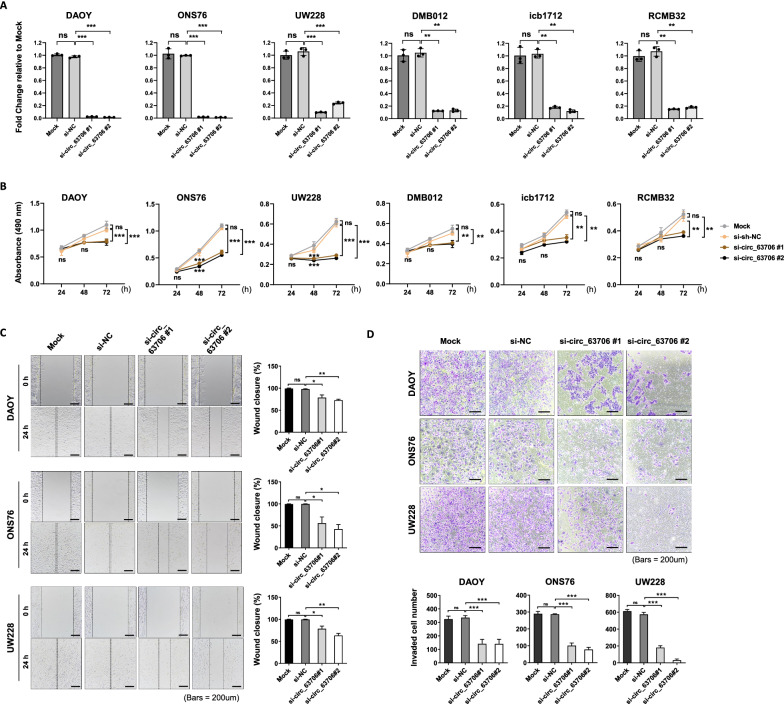
Inhibition of *circ_63706* suppresses SHH MB cell proliferation, migration, and invasion in vitro. **A** *circ_63706* expression was detected in DAOY, ONS76, UW228, DMB012, icb1712, and RCMB32 cells with si-*circ_63706* or NC. **B** MTS assays were performed to assess proliferation in DAOY, ONS76, UW228, DMB012, icb1712, and RCMB32 cells with si-*circ_63706* or NC. **C** Cell migration was assessed by a wound healing assay in DAOY, ONS76, and UW228 cells with si-*circ_63706* or NC. **D** Cell invasion was assessed by transwell assays and DAOY, ONS76, and UW228 cells with si-*circ_63706* or NC. Scale bar, 200 μm. Data shown are mean ± SD. *p < 0.05, **p < 0.01, ***p < 0.001

To investigate the function of *circ_63706*, we used two individual siRNAs to inhibit *circ_63706* expression in DMB012, icb1712, and RCMB32 SHH MB PDXs and DAOY, ONS76, and UW228 SHH MB cell lines. Transfection with siRNAs targeting *circ_63706* significantly and almost completely abolished *circ_63706* expression compared with controls (si-NC) in these SHH MB cell lines and PDXs (*p* < 0.01, Fig. 3A) without affecting host gene expression (*PCNT*), (Additional file 3: Fig. S12). *circ_63706* knockdown significantly inhibited cell proliferation in all SHH MB cell lines and PDXs (*p* < 0.01, Fig. 3B). Furthermore, *circ_63706* knockdown significantly inhibited SHH cell migration and invasion (*p* < 0.01, Fig. 3C, D). Conversely, restoring circRNA levels rescued the knockdown phenotype (Additional file 3: Fig. S13).

To gain further insights into the functional significance of *circ_63706*, gene expression was measured by RNA-seq in DAOY cells treated with either si-NC or si-*circ-63706*. Among 735 genes with a significant change in expression (FDR < 0.05), 340 genes were upregulated and 395 genes were downregulated in cultured DAOY cells treated with si-*circ-63706* (Additional file 3: Fig. S14A). Ingenuity Pathway Analysis (IPA) revealed that *circ_63706* knockdown preferentially affected genes associated with cell proliferation and apoptosis (Additional file 3: Fig. S14B). Of note, *circ_63706* knockdown downregulated genes contributing to important cancer pathways including *RBBP4*, *TGFA*, *E2F1*, and *HRAS* (Additional file 3: Figs. S14 and 15).

### *Circ_63706* regulates tumor formation in mouse intracranial xenografts

**Fig. 4:**
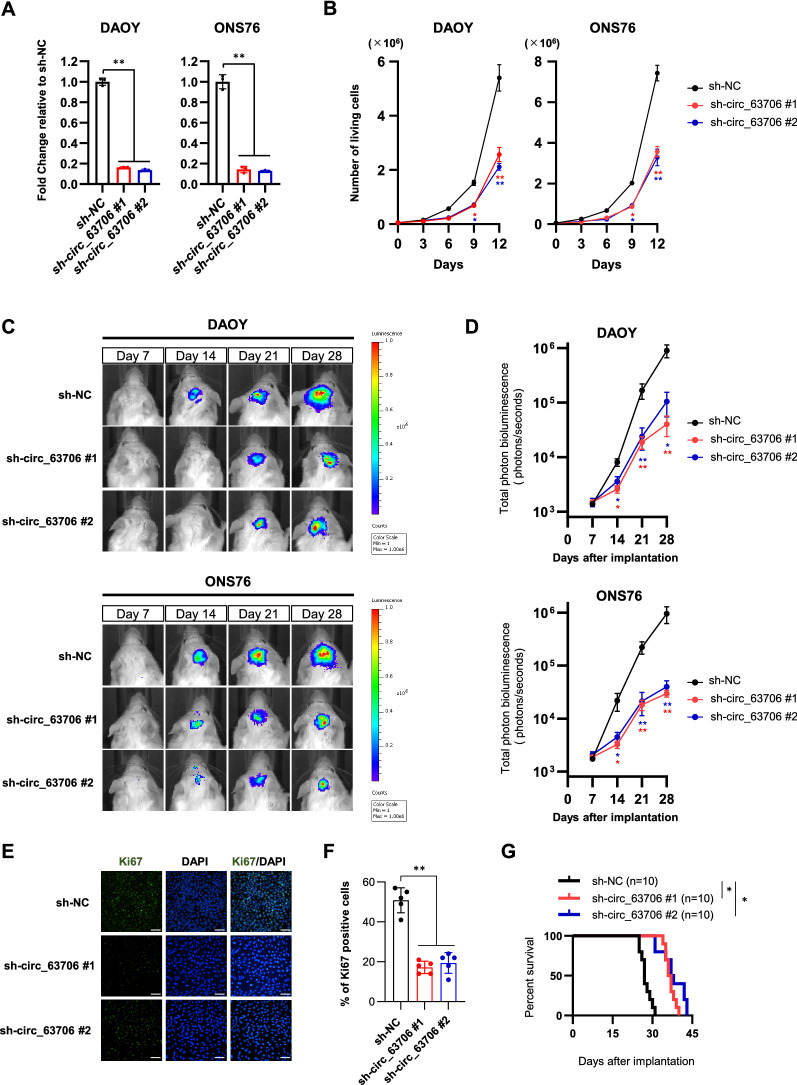
*Circ_63706* promotes tumorigenesis and growth of SHH MB cell in vivo. **A** Expression of *circ_63706* in DAOY and ONS76 control (*sh-NC*) and DAOY and ONS76 with *circ_63706*-knockdown (sh-*circ_63706* #1 and #2) cells. Relative expression to *sh-NC* is indicated on the y-axis. **B** Cell viability assays performed with DAOY and ONS76 control (*sh-NC*) and DAOY and ONS76 with *circ_63706*-knockdown (sh-*circ_63706* #1 and #2) cells. Points represent the mean ± SD of three biological replicates. **C** DAOY and ONS76 control (*sh-NC*) and DAOY and ONS76 with *circ_63706*-knockdown (sh-*circ_63706* #1 and #2) cells expressing luciferase were implanted into the cerebellums of NOD-SCID mice, and tumor formation was assessed by bioluminescence imaging. Changes in bioluminescent signal were examined weekly after tumor implantation. **D** Quantification of total photon counts from mice implanted with DAOY and ONS76 control (*sh-NC*) and DAOY and ONS76 with *circ_63706*-knockdown (sh-*circ_63706* #1 and #2) cells. n = 5. **E** Ki67 staining of xenograft tumor sections. Nuclei are stained with DAPI. Scale bars, 50 μm. Quantification of Ki67-positive cells are shown in (**F**). *p < 0.05, Student’s *t*-test. **G** Overall survival was determined by Kaplan–Meier analysis, and the log-rank test was applied to assess the differences between groups. *p < 0.05, Mantel–Cox log-rank test

To evaluate the effect of *circ_63706* on tumor growth in vivo, we established intracranial MB xenografts in NOD-SCID mice. We knocked down *circ_63706* in DAOY and ONS76 SHH cells using a lentivirus with a luciferase reporter (Fig. 4A, B). Weekly evaluation of tumor growth by bioluminescence imaging revealed significantly smaller tumors in mice transplanted with *circ_63706*-knockdown DAOY and ONS76 cells than in mice transplanted with control cells (n = 5, *p* < 0.05, Fig. 4C, D).

On day 28, Ki67 immunofluorescence analysis in tissue sections of excised tumors showed reduced cell proliferation in *circ_63706*-knockdown ONS76 tumors (*p* < 0.01, Fig. 4E, F). Kaplan–Meier plots demonstrated that the group transplanted with *circ_63706*-knockdown cells had significantly prolonged survival compared with control (Fig. 4G). Together, these results demonstrate that *circ_63706* regulates tumor growth in vivo and may function as an oncogene.

### *Circ_63706* depletion enhances lipid oxidation and bioactivity and reduces total triglycerides

**Fig. 5:**
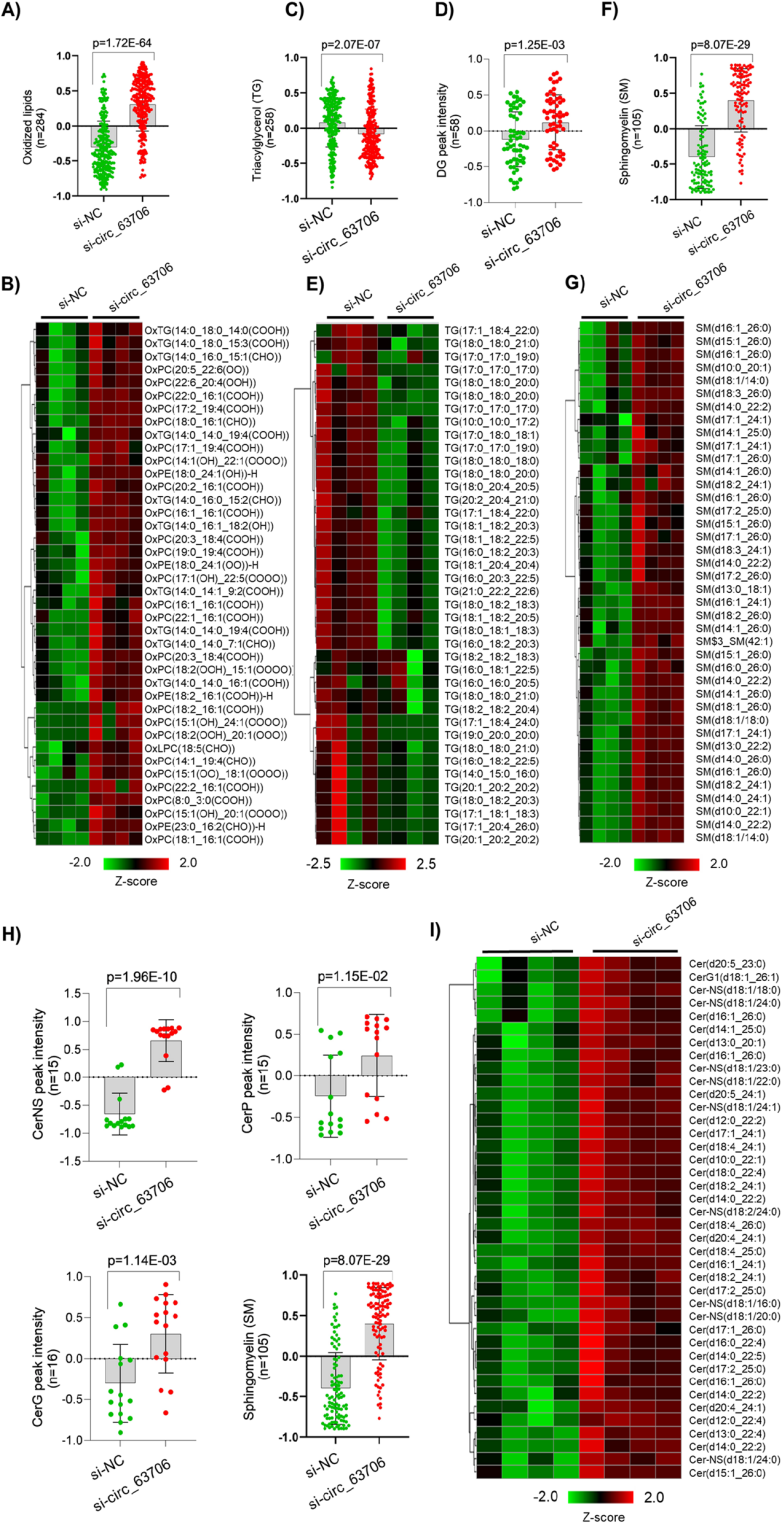
*Circ_63706* suppresses lipid oxidation and membrane components. The box plot presents total oxidized lipids in *circ_63706* knockdown cells (**A**) and the heatmap represents the top 40 oxidized lipid molecules in *circ_63706* knockdown cells (**B**). The box plot presents total TG (**C**) and DG (**D**) lipids in *circ_63706* knockdown cells, and the heatmap represents the top 40 TG molecules in *circ_63706* knockdown cells (**E**). The box plot presents total SMs (**F**) and the heatmap represents the top 40 SM molecules in *circ_63706* knockdown cells (**G**). The box plot presents total ceramide and its subclasses in *circ_63706* knockdown cells (**H**) and the heatmap represent the top 40 ceramides and its subclasses in *circ_63706* knockdown cells compared with control (**I**)

Lipid metabolism is a key factor in tumor cell proliferation and growth. As *circ_63706* promoted tumor growth in vitro and in vivo, we further explored the underlying molecular basis in *circ_63706-*knockdown cells using an untargeted lipidomics approach. Interestingly, *circ_63706* knockdown globally enhanced lipid oxidation and downregulated total triglycerides (TGs) (Fig. 5). Lipid oxidation induces toxicity in cancer cells and ultimately leads to cell death. Using ultra-high-pressure liquid chromatography high-resolution mass spectrometry (UHPLC-HRMS)-based global lipidomics, we found that total oxidized lipid (n = 284) was significantly higher in *circ_63706*-knockdown cells compared with controls (Fig. 5A), suggesting that *circ_63706* may suppress fatty acid oxidation in medulloblastoma. We also identified the top 50 upregulated oxidized lipids and found that both glycerophospholipids (PC and PE) and glycerolipids (DG and TG) were mainly oxidized (Fig. 5B). Lipid oxidation in *circ_63706-*knockdown cells may impact other key lipid molecules critical for cancer cell proliferation and growth, such as TGs. Importantly, DGs (n = 58), the breakdown product of TGs, were slightly elevated, whereas total TGs (n = 258) were significantly reduced in *circ_63706* KO cells, suggesting impaired glycerolipid metabolism that may interfere with cellular proliferation (Fig. 5C, D). Furthermore, both saturated and unsaturated fatty acids containing TGs were highly affected by *circ_63706* knockdown (Fig. 5E). Since *circ_63706* appeared to promote a lipid landscape benefitting tumor cells, we further explored the impact of *circ_63706* on bioactive lipids, which are usually toxic to tumor cells. Sphingolipids, including different ceramides, are known to regulate cancer cell signaling to control tumor growth and survival [30]. Using the global lipidomics approach, we found that *circ_63706* markedly suppressed ceramide and sphingolipid production (Fig. 5F–I). Total sphingomyelin (SM; n = 105) was significantly upregulated in *circ_63706* knockdown cells (Fig. 5F). When restricted to the top 40 SMs, their levels were consistently upregulated in *circ_63706* knockdown cells (Fig. 5G). Ceramide accumulates as a bioeffector that mediates cancer cell death. We found that *circ_63706* knockdown cells were significantly enriched for different forms of ceramide including Cer (ceramide) itself and its different subclasses such as CerNS (nitroso-ceramide), CerP (phosphorylated-ceramide), and CerG (glycosylated-ceramide) (Fig. 5H, I). Overall, *circ_63706* may play a critical role in suppressing the accumulation of the bioactive lipid molecules to favor medulloblastoma proliferation and growth.

Fatty acids (FAs) and their biochemistry are increasingly recognized as important in cancer and therapeutic development. We found that *circ_63706* knockdown significantly enriched for active lipid ontologies including sphingosine, sphingomyelin, and ceramide, while *circ_63706* overexpression significantly reduced the active lipid component. Unsaturated fatty acids, specifically fatty acids with 2, 3, and 6 double bonds, were elevated in *circ_63706* knockdown cells. Interestingly, saturated lipids were elevated in *circ_63706*-overexpressing cells (Additional file 3: Fig. S16). Strikingly, there were distinct modulations of FA chain length in *circ_63706* knockdown SHH cells: FAs with 20 or more carbons were significantly reduced and FAs with 18 or fewer carbons were markedly elevated in *circ_63706* knockdown SHH cells. Taken together, our data demonstrate that *circ_63706* promotes FA metabolism in the SHH medulloblastoma subtype.

### Mapping the secondary structure and modeling the 3D structure of *circ_63706*

Characterizing the 3D structures of circRNAs is crucial for understanding their cellular functions such as microRNA sponging and interactions with RNA-binding proteins and other RNAs. The experimental determination of RNA structure is challenging, but circRNA structure determination is especially difficult due to significant overlap between circRNA and linear RNA sequences [22]. There is no experimentally determined circRNA structure currently available. Recent advances in computational modeling based on experimental RNA structure data have allowed RNA structures to be predicted with high accuracy. This information is important for future therapeutic targeting either with antisense oligonucleotides or small molecules that bind to highly conserved secondary structures in circRNAs*.* Therefore, we mapped the secondary structure of the *circ_63706* sequence using MXfold2, which uses deep learning to integrate thermodynamic information to accurately predict secondary structures of newly discovered ncRNAs [34]. This information was used to predict the 3D structure of *circ_63706* with FARFAR2, which uses an RNA fragment assembly method to model RNA structures.

**Fig. 6:**
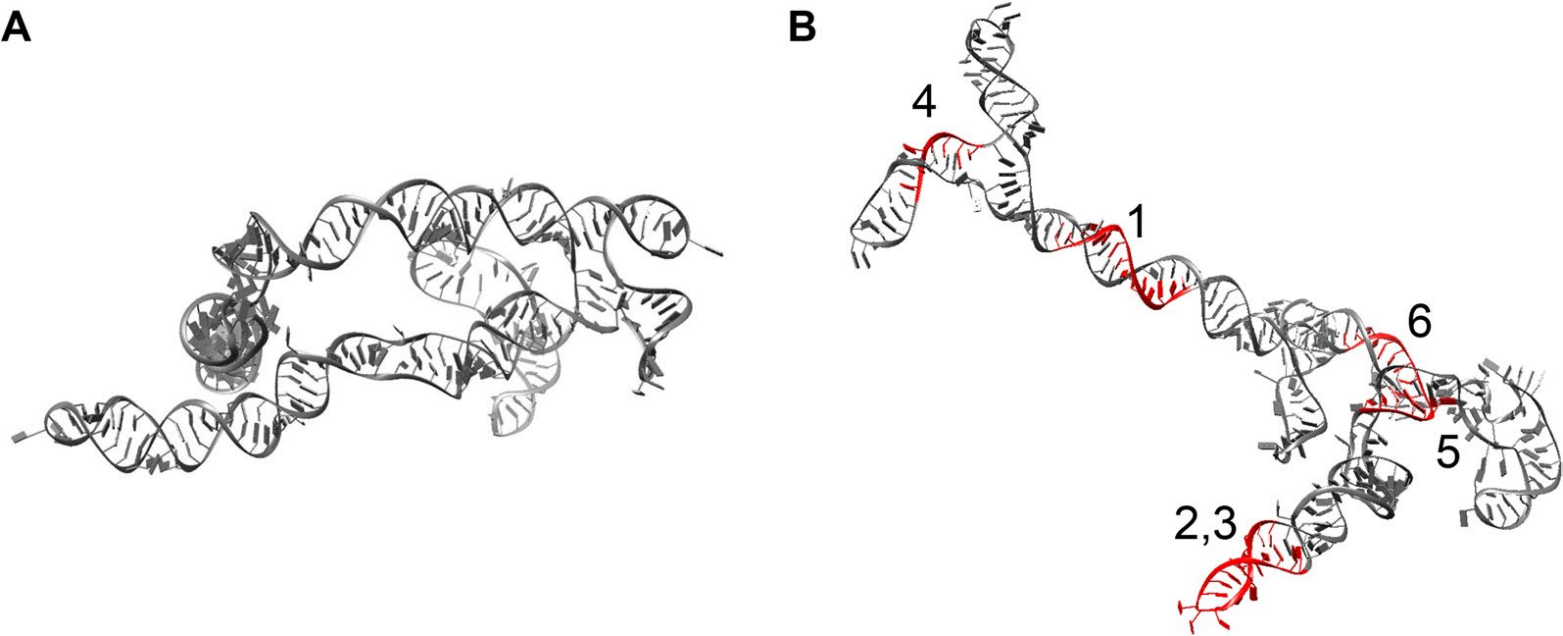
Mapping the secondary structure and modeling the 3D structure of *circ_63706.* The structure of circ_63706 **A** after the minimization of the modeled structure and **B** after 500 ns of MD simulation. Potential miRNA binding sites are highlighted in red and numbered according to the list in Additional file 4: table S6

A 500-ns molecular dynamics simulation of the modeled *circ_63706* showed that the double-stranded pairings were stable, while the overall tertiary structure remained flexible. Figure 6 compares the initial modeled structure with that at the end of the 500 ns simulation. Analysis of the structure with x3DNA-DSSR showed that the *circ_63706* structure has two major dsRNA helix stems with 29 and 19 base pairs, respectively, as well as several other shorter segments ranging from 2 to 9 base pairs. Similarly, the structural features also include three 3-way junctions, one 4-way junction, six hairpin loops, and 12 internal loops. To explore the binding regions of the miRNA, we used the database of 87 mature miRNA human sequences from miRBase paired with the *circ_63706* sequence using IntaRNA. Six top-ranked circRNA-miRNA duplexes, along with their binding energies compared with those within the circRNA structure, are shown in Additional file 4: Table S6. The binding sites in the structure are highlighted in Fig. 6.

## Discussion

To identify medulloblastoma subgroup-specific circRNA biomarkers, we subjected publicly available RNA-seq data to the CIRI2 circular RNA detection pipeline. By applying machine learning and statistical methods, we identified group-specific circRNAs. Among these identified circular RNAs, *circ_63706* (hsa-PCNT_0003, CircAtlas 2.0) was a potential SHH subgroup-enriched molecule, confirmed by qPCR and RNA-FISH of clinical tissue samples.

WNT and SHH MBs generally contain mutations activating those pathways and, aside from rare *TP53*-mutant SHH tumors, are less aggressive than G3 and G4 tumors. Biomarkers for these two groups include immunohistochemical stains for YAP1, nuclear β-catenin, monosomy 6 in WNT tumors, and identifying activating pathway mutations through sequencing [9]. SHH MBs are generally identified by co-expression of GAB1 and YAP1 and by demonstrating activating mutations. Transcriptional or methylation profiling approaches can also distinguish the four groups but are usually not accepted clinical assays.

A recent study identified somatic copy number aberrations (SCNAs) in 1,087 unique MBs [26]. The most common focal copy number gain was a tandem duplication of *SNCAIP*, a gene associated with Parkinson’s disease, exquisitely restricted to group IV alpha. Recurrent translocations of *PVT1*, including *PVT1-MYC* and *PVT1-NDRG1* arising through chromothripsis, were limited to G3 tumors. Numerous targetable SCNAs, including recurrent events targeting TGF-beta signaling in G3 and NF-kappaB signaling G4, are attractive candidate molecular markers. Circular RNAs can act as tumor suppressors or oncogenes, but little is known about their role in MBs. In a recent study, Lv et al. [25] selected four paired normal cerebellum and medulloblastoma tissue samples for sequencing and identified 33 differentially-expressed circRNAs in medulloblastoma tissues. Two of these circRNAs, circular-spindle and kinetochore associated complex subunit 3 (*circ-SKA3*) and *circ-DTL*, promoted the malignant phenotype of medulloblastoma when upregulated. Significantly higher expression of *circ-SKA3* has also been reported in medulloblastoma compared with normal tissues [25].

Ours is the first comprehensive in silico analysis of circular RNAs in medulloblastoma and the first detailed characterization of one circular RNA (*circ_63706*; hsa-PCNT_0003, CircAtlas 2.0) in medulloblastoma patient tissues. The availability of specific classes of lipid is critical for successful cancer cell proliferation. TGs are critical to cell membrane homeostasis, lipid droplet formation, and signaling through lipid rafts for oncogenesis [2]. We demonstrated that *circ_63706* is important for modulating TG and DG levels in SHH cells (Fig. 5 and Additional file 3: Fig. S15). Since cell membranes and their major components such as lipid rafts harboring signaling receptors cannot function properly without the appropriate DG-TG distribution [1], *circ_63706* may play an important role in regulating the lipid component of cell or organelle membranes and, consequently, facilitating the malignant MB phenotype. Indeed, we observed that *circ_63706* overexpression positively modulates membrane components, plasmalogen, and glycerolipids and glycerophospholipids (Additional file 3: Fig. S15). An accumulation of oxidized lipid is toxic to cancer cells through induction of cell death through ferroptosis [35]. Here we revealed that *circ_63706* significantly decreases oxidized lipids in SHH cells and could be responsible for the observed increased MB cell proliferation. *circ_63706* globally affects bioactive lipids and may be a potential therapeutic target. Cancer cells harbor differential FA saturations and chain lengths, and this fatty acid biochemistry and its perturbation can form the basis for therapeutic targets [17]; indeed, *circ_63706* significantly modulated both fatty acid saturation and chain length (Additional file 3: Fig. S16). Since fatty acids are the building blocks of major lipids and their dysregulation plays critical roles in reprogramming cancer cell metabolism [10], this is a potential mechanisms by which *circ_63706* drives cancer cell proliferation, growth, and metastasis, as *circ_63706* overexpression drive increase cell proliferation (Additional file 3: Fig. S13).

Our molecular dynamics simulation of the modeled *circ_63706* suggest that several miRNAs can bind to *circ_63706* (Additional file 4: table S6). *hsa-miR-26b-3p* is a known tumor suppressor that regulates cellular proliferation, growth, and metastasis in osteosarcoma [5] and breast cancer [20]. Similarly, *hsa-miR-103a-2-5p* is a tumor suppressor in prostate cancer [4] and in squamous cell carcinoma of the tongue [23]. Interestingly, circRNA *circ_0007142* can function as an miRNA sponge and inhibit *hsa-miR-103a-2-5p,* promoting proliferation in colorectal cancer [44]. Also, sequestering of *hsa-miR-103a-2-5p* by another circRNA *circ_0087293* (circRNA-SORE) acting as a sponge promotes drug resistance in hepatocellular carcinoma [39]. We detected *hsa-miR-22-5p* as another potential *circ_63706*-binding miRNA, which is reported as a suppressor of breast cancer metastasis [14] and can reverse drug resistance in colon cancer [18].

Overall, this study provides several important insights into novel molecular signatures of medulloblastoma subgroups for diagnostic and therapeutic purposes.

## Supplementary Information

**Additional file 1**: Supplementary File.

**Additional file 2**: Supplementary Methods.

**Additional file 3**: Supplementary Figures.

**Additional file 4**: Supplementary Tables.

## Data Availability

RNA-seq data described in the manuscript is accessible at NCBI GEO accession number GSE216687.
