# Supplementary material for: The oncogenic circular RNA circ_63706 is a potential therapeutic target in sonic hedgehog-subtype childhood medulloblastomas
Source: Acta Neuropathol Commun. 2023 Mar 10;11:38. doi: 10.1186/s40478-023-01521-0 (PMC10007801; doi:10.1186/s40478-023-01521-0)
Supplement: Supplementary file 1 — Additional file 1: Supplementary File. [file 40478_2023_1521_MOESM1_ESM.docx]

**Supplemental Methods**

**Circular RNA identification and quality control**

　　The ultra-fast FASTQ preprocessor package *fastp* ^1^ was used for quality control, adapter trimming, and filtering low-quality FASTQ read data. Filtered FASTQ files were mapped to the human ribosomal DNA complete repeating unit (GenBank: U13369.1) using *bowtie2* ^2^ read aligner; this allowed us to remove ribosomal RNA (rRNA) from reads. Unmapped reads were filtered and extracted using a combination of *samtools* and *bedtools*. Human reference DNA and gene annotation files were downloaded from Ensembl v102. rRNA-depleted reads were aligned to the human reference genome and SAM files were generated with the BWA-MEM aligner tool ^3^. The CIRI2 ^4^ workflow was used for circular RNA detection from aligned SAM files. Although many reliable algorithms are available to detect circRNAs, we chose CIRI2 based on a recent comparative study of eleven circRNA detection algorithms ^5^, which emphasized the reliability and performance of the CIRI2 algorithm. The circRNAs identified by CIRI2 were aggregated to count matrix format using the circM.py script ^5^.

**Identification of differentially expressed circRNAs**

　　The CIRI2 algorithm ^4^ detected 79,099 circRNA loci across all 175 medulloblastoma samples. Samples with <1000 circRNAs or very low counts across samples were discarded. The completed count matrix contained 126 medulloblastoma samples and 8925 highly abundant circRNAs. The *limma-voom* package is fast, flexible, maintains the false discovery rate (FDR) at or below the nominal rate, and employs empirical Bayes smoothing of gene-wise standard deviations suited to RNA-seq data ^6,7^. We used this package for differential expression analysis of filtered RNA-seq data ^7^, and p-values were adjusted using the Benjamini and Hochberg method for controlling the FDR.

**3D structural modeling and molecular dynamics simulation of *circ_63706***

　　The Rosetta prediction tool FARFAR2 ^8-10^ was used to model a 3D structure of *circ_63706* using the secondary structure information obtained with *MXfold2* ^11^. The top-ranked 3D structure of *circ_63706* was further relaxed with molecular dynamics (MD) simulations. For this, the simulation system was set up using the solution builder tool in CHARMM-GUI ^12-14^. The modeled structure had the 5’ and 3’ ends close to each other, and this allowed modeling of the circular structure by linking the ends with a patch. The structure was solvated with TIP3 water at a salt concentration of 0.15 mM. The final, solvated system contained ~300,000 atoms. After a 10,000-step minimization, the system was equilibrated for 2 ns, followed by a 500 ns production run under constant pressure of 1 atm and constant temperature of 303.15 K with 2 fs time step. The simulation was performed with a Charmm36m force field ^15,16^ and NAMD2.14 ^17^ simulation package. Trajectory was analyzed and visualized with Visual Molecular Dynamics (VMD) ^18^ and UCSF Chimera ^19^. Finally, the pairwise binding of different miRNAs to the circRNA target was predicted with IntaRNA ^20^.

**Lipidomic analysis**

　　First, cell pellets were washed three times with 40 mM ammonium formate buffer before re-suspension in 50 µL 5 mM ammonium acetate. Homogenization was performed three times with Zirconia beads on a bead beater for 30 s each time with 15 min of incubation on ice between each bead beater treatment. Protein concentrations were measured, and cell homogenates were normalized to a protein concentration of 300 ug/mL. The 300 ug/mL sample homogenates were then transferred to 15 mL glass tubes for global lipidomic extraction as previously described ^21^. Briefly, 20 μL of internal standard mixture containing lysophosphatidylcholine (LPC 17:0), phosphatidylserine (PS 14:0/14:0), phosphatidylcholine (PC 17:0/17:0), phosphatidylglycerol (PG 14:0/14:0), phosphatidylethanolamine (PE 15:0/15:0), sphingomyelin (SM d18:1/17:0), ceramide (Cer d18:1/17:0), diacylglycerol (DG 14:0/14:0), triacylglycerol (TG 15:0/15:0/15:0), bis(monacyl-glycero)phosphate (BMP 14:0 (S,R), and Lyso SM(d17:1), each at 100 ppm in 2:1 chloroform:methanol, was added. Except for TG, all other lipid standards were purchased from Avanti Polar Lipids (Alabaster, AL), while TG was purchased from Sigma-Aldrich (St. Louis, MO). Extraction was performed by adding ice cold 4:2:1 chloroform:methanol:water (v:v:v), and the organic phase was collected using low speed centrifugation at 3500 rpm for 10 min at 4°C. Collected organic phase was dried down under nitrogen flow and reconstituted in 50 μL of isopropanol plus 1 μL of injection standard mixture containing LPC(19:0), PC(19:0/19:0), PG(17:0/17:0), PE(17:0/17:0), PS(17:0/17:0), and TG(17:0/17:0/17:0), each at 100 ppm in 2:1 chloroform:methanol. For the sample run, solvent blanks, extraction blanks (without internal standard), and neat quality controls (lipid internal standards mixtures), pooled samples per group (5 µL of each sample) were also prepared for evaluation of extraction and data collection efficiency.

Chromatographic separation for lipidomics was achieved on a Waters Acquity C18 BEH column maintained at 50°C (2.1 × 100 mm, 1.7 μm particle size, Waters, Milford, MA). The mobile phases consisted of solvent A (60:40 acetonitrile:water) and solvent B (90:8:2 isopropanol:acetonitrile:water), both with 10 mM ammonium formate and 0.1% formic acid. The gradient elution was ramped from 20% D to 98% D with a 0.5 mL/min flow rate over 17.00 min followed by 3.00 min column flush and re-equilibration. The flow rate was 500 μL/min. Samples were analyzed in positive and negative electrospray ionization on a Thermo Scientific Q-Exactive mass spectrometry with Dionex Ultimate 3000 UHPLC (Thermo Scientific, San Jose, CA). Data-dependent (ddMS2-top5) MS/MS and AIF (All-ion fragmentation) data were obtained on pooled samples per group for identification purposes.

For lipidomics data analysis, LipidMatch Flow was used for file conversion, peak selection (implementing MZMine ^22^), blank filtration, lipid annotation ^23^, and combining positive and negative datasets. LipidMatch Flow was used to annotate ions using data-dependent MS/MS analysis.

**Cell proliferation, migration, and invasion assays**

　　DAOY, ONS76, and UW228 cells were placed in 96-well plates at 3 × 10^3^ cells per well for the cell proliferation assay and incubated overnight. After transfection with two individual *circ_63706* siRNAs and a negative control siRNA, cell viability was measured using the MTS assay every 24 h with the CellTiter Cell Proliferation Reagent Kit (Promega) according to the manufacturer’s protocol. Cells were seeded into 12-well plates for the cell migration assay and incubated overnight. After 48 hours of transfection, a 200 μL pipette tip was used to make a wound in the cells. After incubation for 0 and 24 hours, the wound area was monitored with a microscope (10× objective) and the migration distance calculated. 24-well Transwell chambers with 8-μm pore size (Corning, Corning, NY) were used for the cell invasion assay. 3 × 10^4^ transfected cells were transferred in serum-free medium into the upper chamber of an insert with Matrigel (1:10 ratio), and medium supplemented with 10% FBS was added to the lower chamber. After incubation for 36 h, cells that had invaded through the other side of the membrane surface were fixed with methanol and stained with 0.5% crystal violet (Acros Organics, Thermo Fisher Scientific). Three random fields were imaged and the counted cells under an ECLIPSE Ts2 inverted microscope (Nikon, Tokyo, Japan).

**shRNA-mediated stable** ***circ_63706*-knockdown cell line**

　　The shRNA targeting *circ_63706* was packaged into lentivirus particles and 293T cells using Lenti-X™ Packaging Single Shots (cat. no. 631278; Takara Bio, Inc., Shiga, Japan), according to the manufacturer’s instructions. For the lentivirus infection, we incubated cells with viral supernatant for 24 hours, followed by puromycin selection until drug-resistant colonies became visible. The following *circ_63706* sequences were targeted (#1): CAGCTGGAGACCCTGAAGGAA, (#2): ACAGCTGGAGACCCTGAAGGA, and (CTRL): TTCTCCGAACGTGTCACGTTT.

**Overexpression of *circ_63706* in medulloblastoma cells**

　　Plasmid *circ_63706* cDNA was constructed by introducing the upstream intron (~800 bp), the downstream intron fragment (the reverse complementary upstream intron sequence, ~800 bp), as well as the sequence for *circ_63706* circularization (RNA sequence lacking the shRNA targeting *circ_63706*) into pcDNA4. DAOY and ONS76 cells were transfection with pcDNA4-*circ_63706* using Lipofectamine 3000 (Thermo Fisher Scientific) according to the manufacturer’s instructions. Cells were collected after transfection for RNA isolation and cell proliferation assay.

**Supplemental References**

**1.** Chen S, Zhou Y, Chen Y, Gu J. fastp: an ultra-fast all-in-one FASTQ preprocessor. *Bioinformatics.* 2018; 34(17):i884-i890.

**2.** Langmead B, Salzberg SL. Fast gapped-read alignment with Bowtie 2. *Nat Methods.* 2012; 9(4):357-359.

**3.** Li H, Durbin R. Fast and accurate short read alignment with Burrows-Wheeler transform. *Bioinformatics.* 2009; 25(14):1754-1760.

**4.** Gao Y, Zhang J, Zhao F. Circular RNA identification based on multiple seed matching. *Briefings in Bioinformatics.* 2018; 19(5):803-810.

**5.** Hansen TB. Improved circRNA Identification by Combining Prediction Algorithms. *Frontiers in Cell and Developmental Biology.* 2018; 6(20).

**6.** Seyednasrollah F, Laiho A, Elo LL. Comparison of software packages for detecting differential expression in RNA-seq studies. *Brief Bioinform.* 2015; 16(1):59-70.

**7.** Law CW, Chen Y, Shi W, Smyth GK. voom: Precision weights unlock linear model analysis tools for RNA-seq read counts. *Genome Biol.* 2014; 15(2):R29.

**8.** Watkins AM, Rangan R, Das R. FARFAR2: improved de novo rosetta prediction of complex global RNA folds. *Structure.* 2020; 28(8):963-976. e966.

**9.** Watkins A, Das R. RNA 3D modeling with FARFAR2, online. *bioRxiv.* 2020.

**10.** Lyskov S, Chou F-C, Conchúir SÓ, et al. Serverification of molecular modeling applications: the Rosetta Online Server that Includes Everyone (ROSIE). *PloS one.* 2013; 8(5):e63906.

**11.** Sato K, Akiyama M, Sakakibara Y. RNA secondary structure prediction using deep learning with thermodynamic integration. *Nature communications.* 2021; 12(1):1-9.

**12.** Jo S, Kim T, Iyer VG, Im W. CHARMM‐GUI: a web‐based graphical user interface for CHARMM. *Journal of computational chemistry.* 2008; 29(11):1859-1865.

**13.** Lee J, Cheng X, Swails JM, et al. CHARMM-GUI input generator for NAMD, GROMACS, AMBER, OpenMM, and CHARMM/OpenMM simulations using the CHARMM36 additive force field. *Journal of chemical theory and computation.* 2016; 12(1):405-413.

**14.** Brooks BR, Brooks III CL, Mackerell Jr AD, et al. CHARMM: the biomolecular simulation program. *Journal of computational chemistry.* 2009; 30(10):1545-1614.

**15.** Huang J, MacKerell Jr AD. CHARMM36 all‐atom additive protein force field: Validation based on comparison to NMR data. *Journal of computational chemistry.* 2013; 34(25):2135-2145.

**16.** Huang J, MacKerell Jr AD. Force field development and simulations of intrinsically disordered proteins. *Current opinion in structural biology.* 2018; 48:40-48.

**17.** Phillips JC, Braun R, Wang W, et al. Scalable molecular dynamics with NAMD. *Journal of computational chemistry.* 2005; 26(16):1781-1802.

**18.** Humphrey W, Dalke A, Schulten K. VMD: visual molecular dynamics. *Journal of molecular graphics.* 1996; 14(1):33-38.

**19.** Pettersen EF, Goddard TD, Huang CC, et al. UCSF Chimera—a visualization system for exploratory research and analysis. *Journal of computational chemistry.* 2004; 25(13):1605-1612.

**20.** Mann M, Wright PR, Backofen R. IntaRNA 2.0: enhanced and customizable prediction of RNA–RNA interactions. *Nucleic acids research.* 2017; 45(W1):W435-W439.

**21.** Tan SK, Mahmud I, Fontanesi F, et al. Obesity-Dependent Adipokine Chemerin Suppresses Fatty Acid Oxidation to Confer Ferroptosis Resistance. *Cancer Discov.* 2021; 11(8):2072-2093.

**22.** Pluskal T, Castillo S, Villar-Briones A, Oresic M. MZmine 2: modular framework for processing, visualizing, and analyzing mass spectrometry-based molecular profile data. *BMC Bioinformatics.* 2010; 11:395.

**23.** Koelmel JP, Kroeger NM, Ulmer CZ, et al. LipidMatch: an automated workflow for rule-based lipid identification using untargeted high-resolution tandem mass spectrometry data. *BMC Bioinformatics.* 2017; 18(1):331.
