# Supplementary material for: The oncogenic circular RNA circ_63706 is a potential therapeutic target in sonic hedgehog-subtype childhood medulloblastomas
Source: Acta Neuropathol Commun. 2023 Mar 10;11:38. doi: 10.1186/s40478-023-01521-0 (PMC10007801; doi:10.1186/s40478-023-01521-0)
Supplement: Supplementary file 3 — Additional file 3: Supplementary Figures. [file 40478_2023_1521_MOESM3_ESM.docx]

**Supplementary Figures**

**
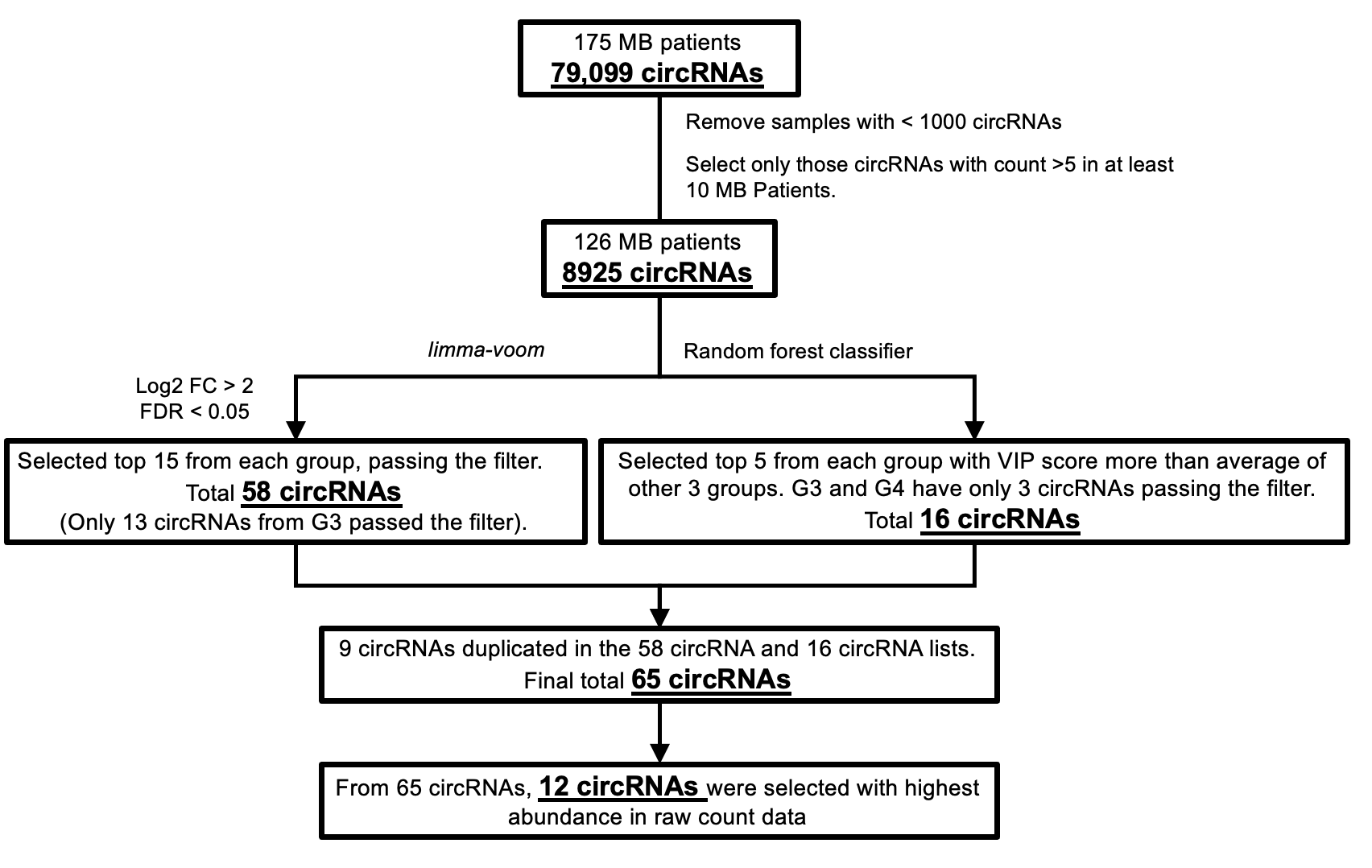
**

**Supplementary Figure 1. Flowchart for identifying differential expression of circRNAs in medulloblastoma subgroups.**

**
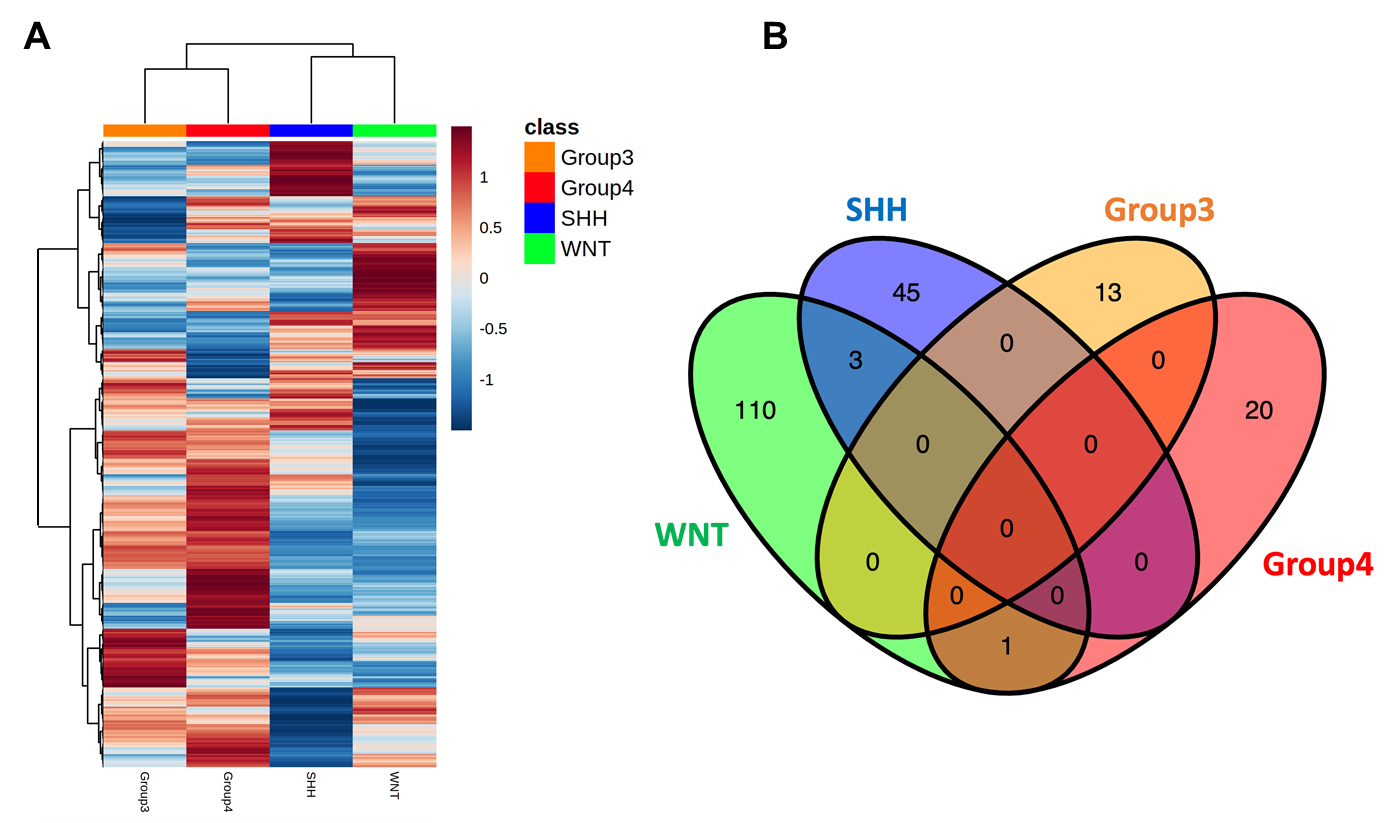
**

**Supplementary Figure 2. 8925 circRNAs separating medulloblastoma subgroups.**

(A) Heatmap cluster plot. (B) Venn diagram of significantly upregulated circRNAs.

**
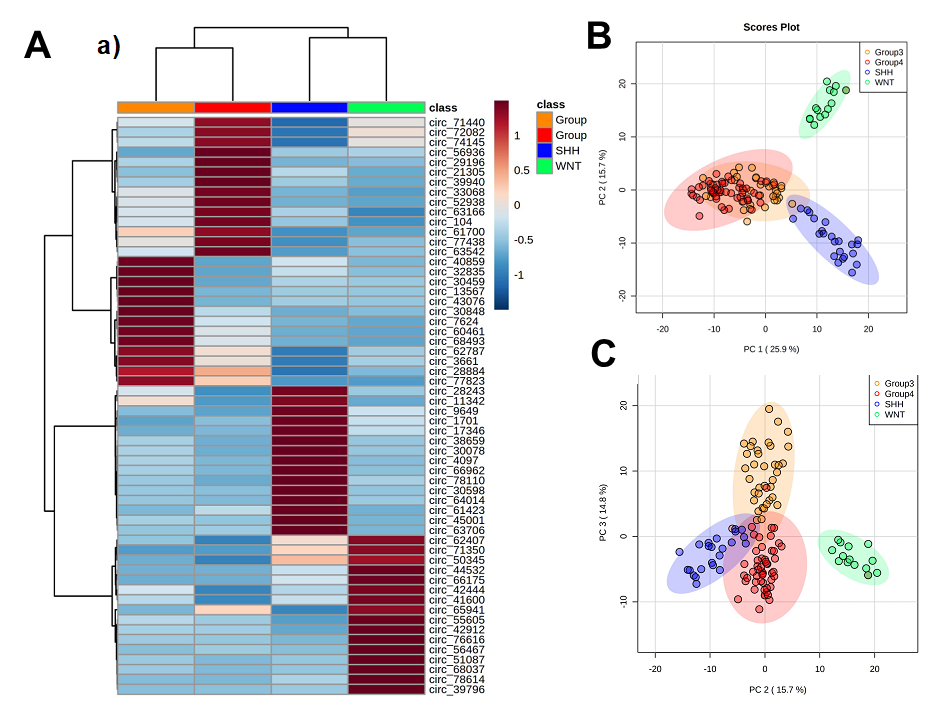
**

**Supplementary Figure 3. 58 circRNAs separating medulloblastoma subgroups.**

(A) Heatmap cluster plot. (B) Principal component analysis (PCA) plot between component-1 and component-2. (C) PCA plot between component-2 and component-3.

**
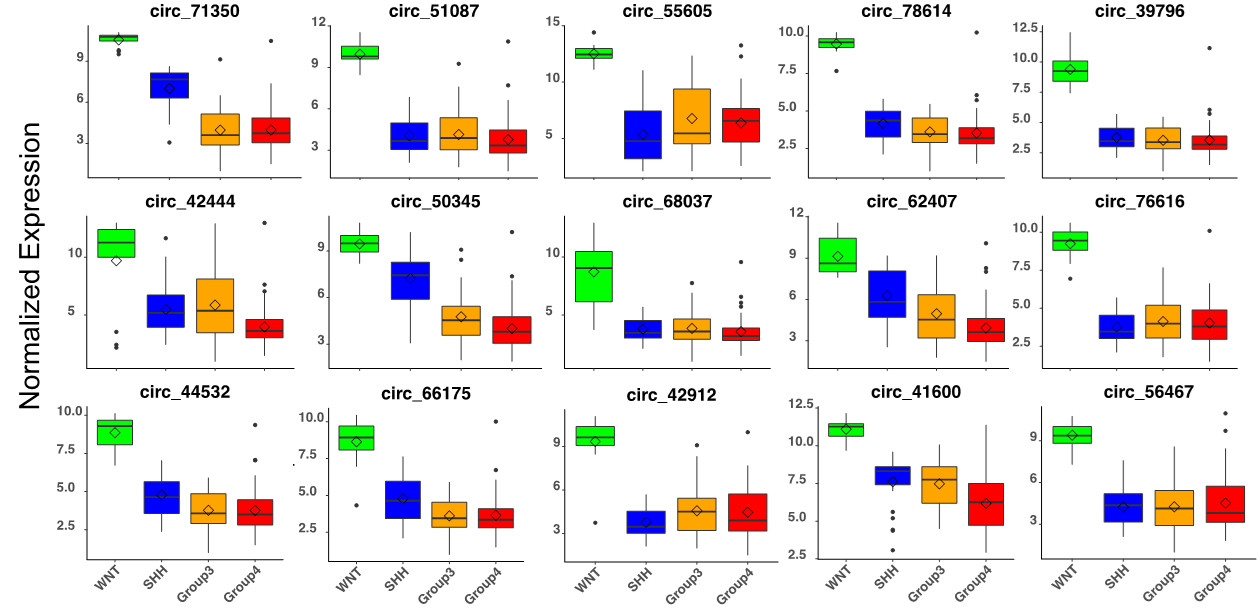
**

**Supplementary Figure 4. Normalized expression of circRNAs highly expressed in the WNT sub-group.**


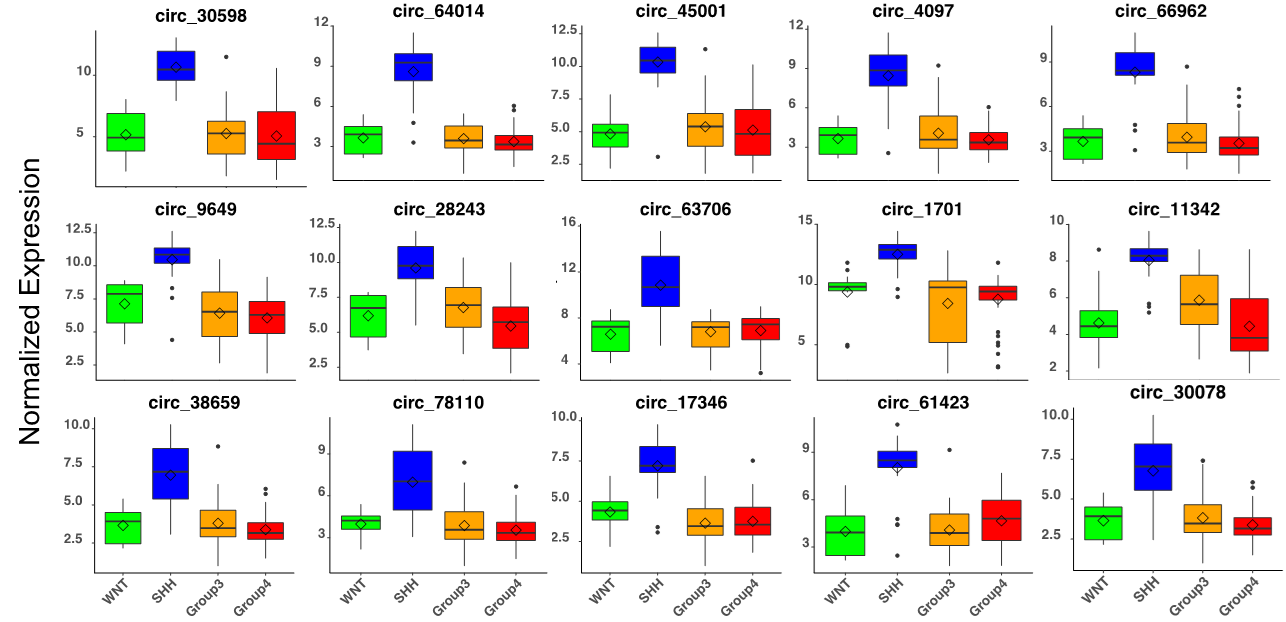


**Supplementary Figure 5. Normalized expression of circRNAs highly expressed in the SHH sub-group.**

**Supplementary Figure 6. Normalized expression of circRNAs highly expressed in the group3 sub-group.**

**Supplementary Figure 7. Normalized expression of circRNAs highly expressed in the group4 sub-group.**

**
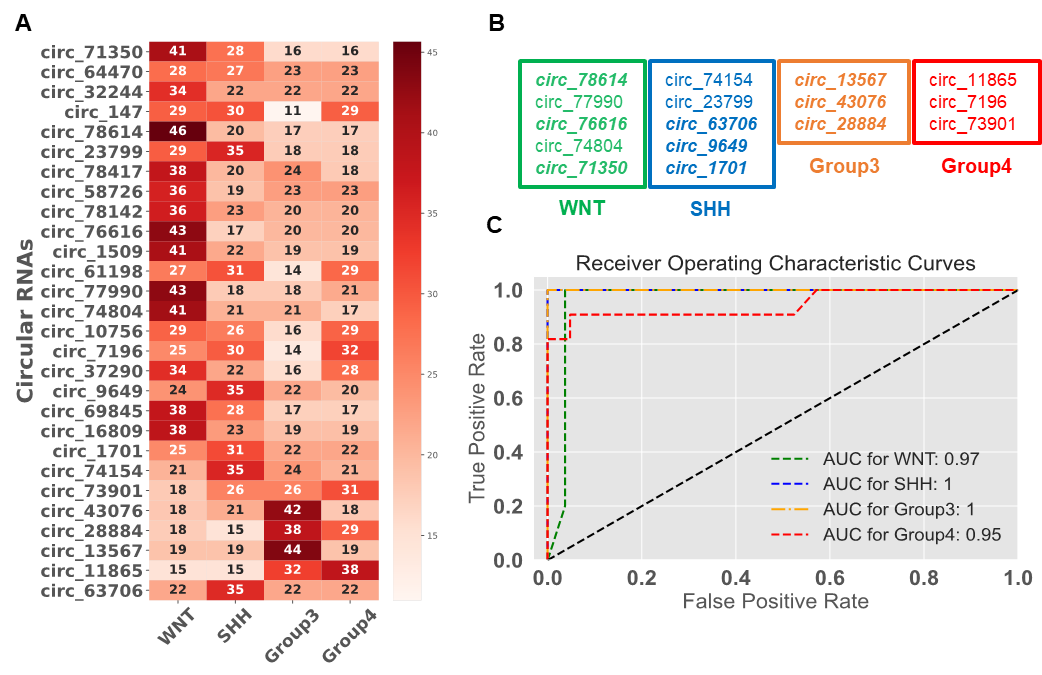
**

**Supplementary Figure 8. Random forest (RF) classification to classify crucial medulloblastoma group-specific circRNAs.**

(A) Heatmap plot of the coefficients of important circRNAs for the classification model. (B) Significant circRNAs predicted by the RF model. The bold and italicized ones are also present in the top 58 significant MD group-specific circRNAs identified by DE analysis. (C) Evaluation of the classification model using ROC curves.

**
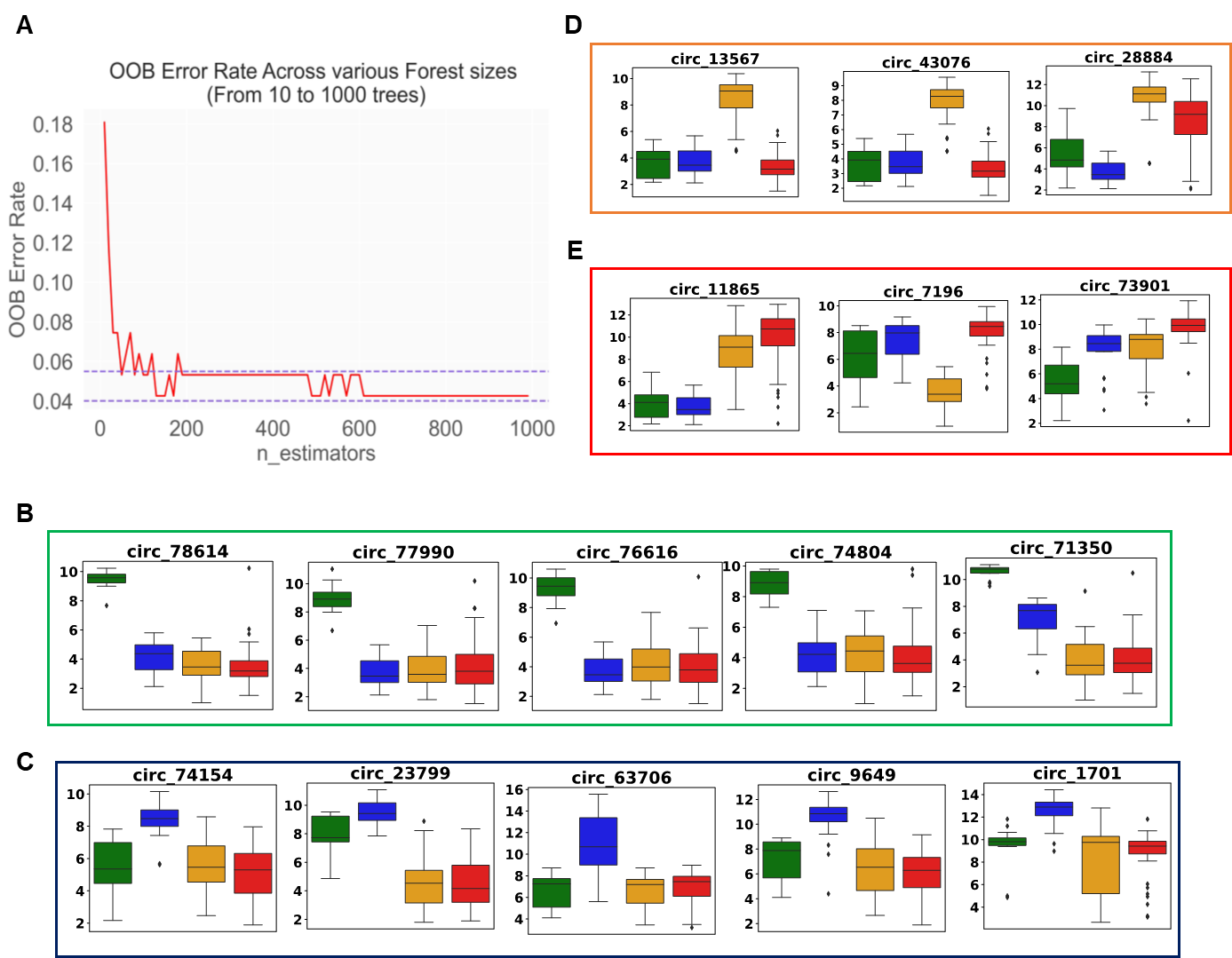
**

**Supplemental Figure 9. Random forest classifier to identify significant features of group-specific MBs.**

(A) Out-of-bag (OOB) error rate for selection number of forests for the random forest classifier. Expression of marker circRNAs in WNT (B), Group 3 (C), SHH (D), and Group 4 (E).


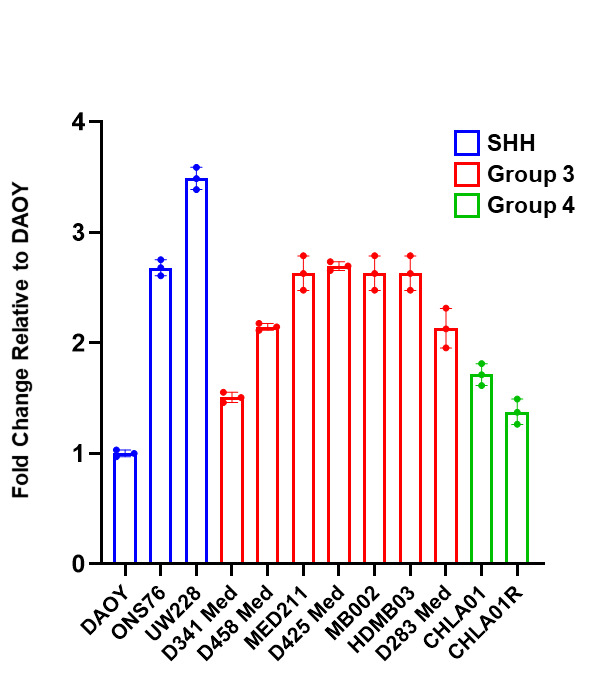


**Supplemental Figure 10. qRT-PCR analysis of *circ_63706* in MB cell lines.**

qRT-PCR analysis showing the distribution of normalized expression values of *circ_63706* in MB cell lines of group 3, group 4, and SSH MBs. Values indicate fold-change relative to DAOY.


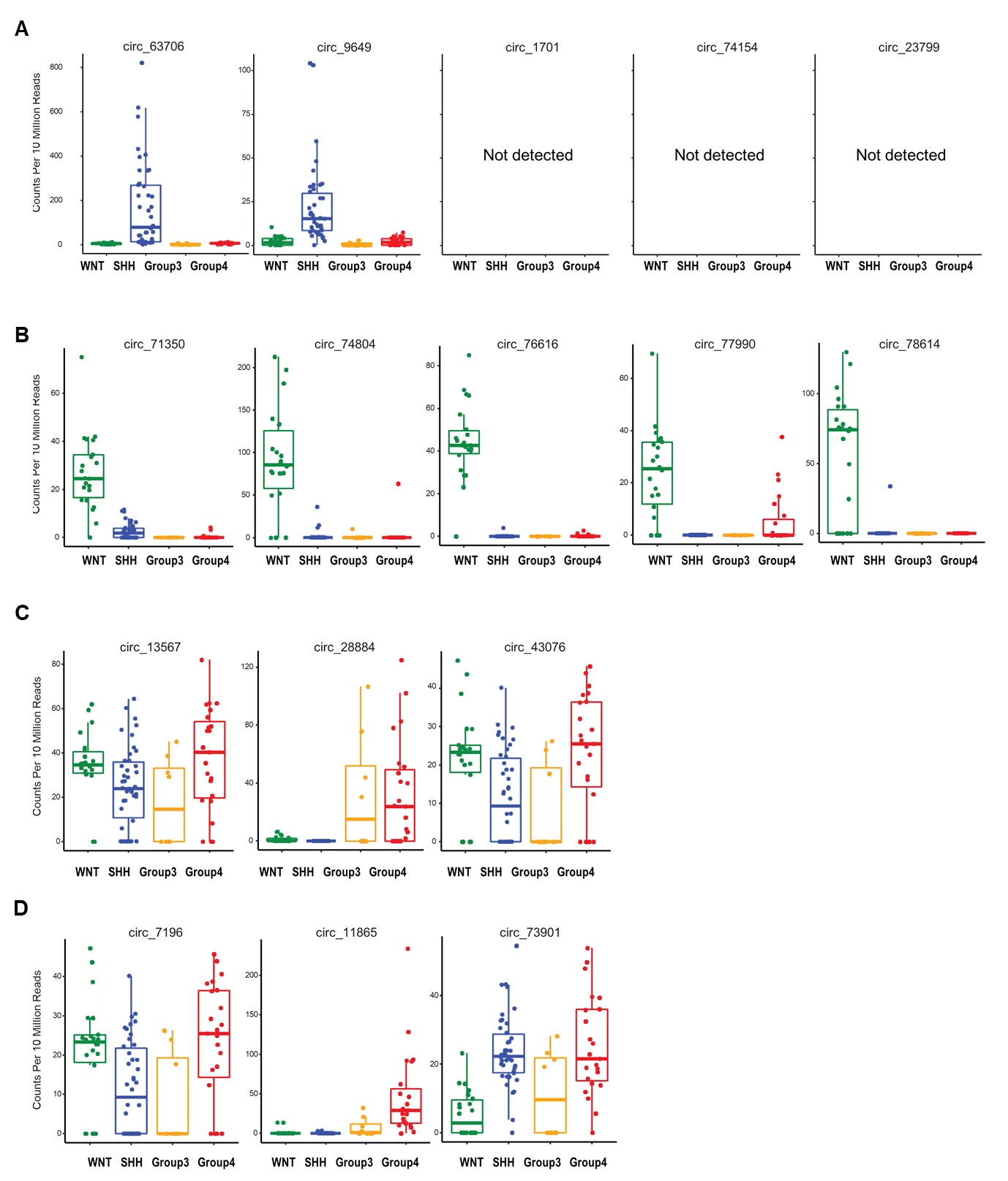


**Supplemental Figure 11. Expression of 16 subgroup-specific circRNAs in St. Jude Cloud database**

Boxplot showing distribution of normalized expression values of 16 subgroup-specific circRNAs in each patient subgroup. (A) SHH-specific circRNAs, (B) WNT-specific circRNAs, (C) group 3-specific circRNAs, (D) group4-specific circRNAs


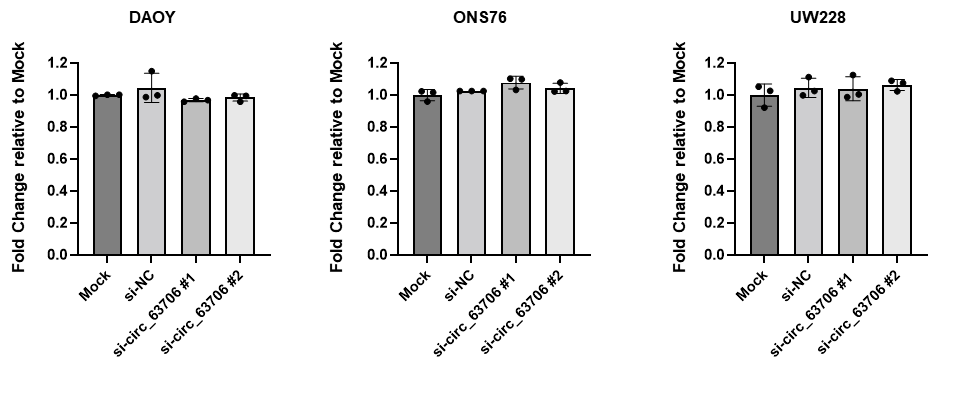


**Supplemental Figure 12. Expression analysis of host *PCNT* gene.**

Expression levels of *PCNT* were examined by qPCR in DAOY, ONS76, and UW228 cells treated with either si-NC or si-*circ-63706*. Relative expression level to mock is indicated in the y-axis (n=3). Error bars indicate S.D.

**
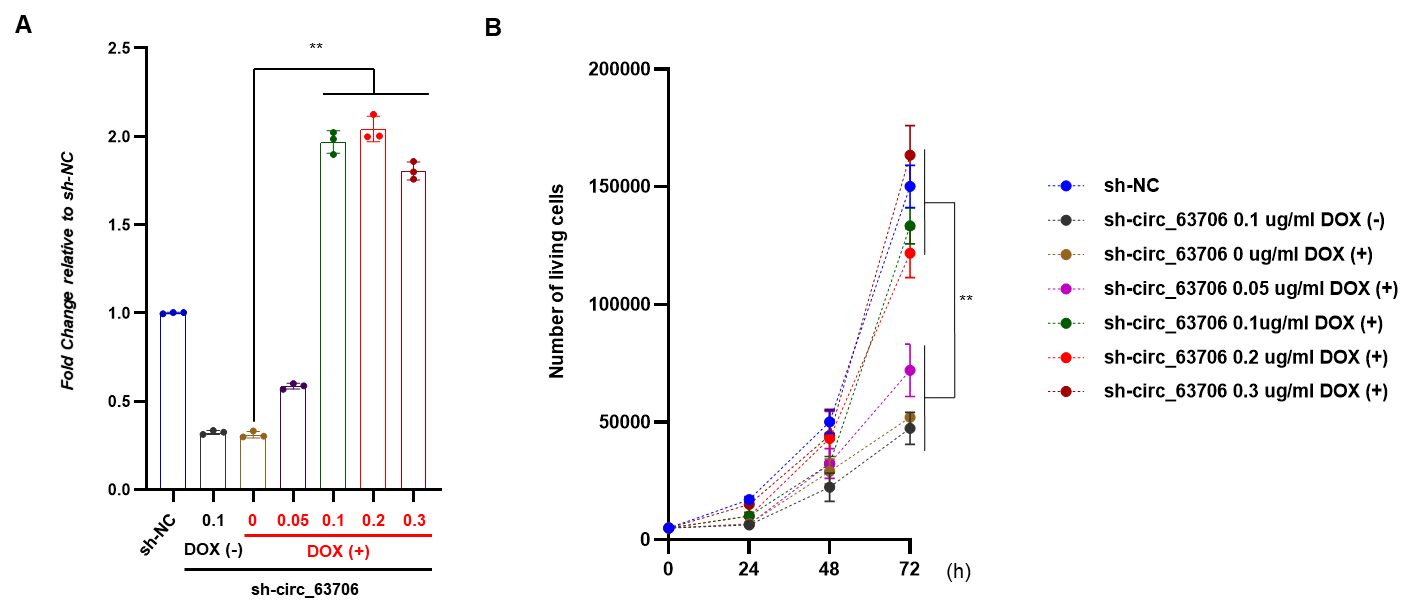
**

**Supplemental Figure 13. Inhibition of cell proliferation was rescued by *circ_63706* overexpression.**

(A) Expression of circ-63706 was examined by qPCR in sh-NC and sh-*circ-63706* stable DAOY cells treated with the plasmid that expresses *circ-63706* (0, 0.05, 0.1, 0.2, 0.3 µg/ml) with or without doxycycline (DOX). The relative expression level to mock is indicated on the y-axis (n=3). Error bars indicate standard deviation. **p< 0.01, Kruskal–Wallis analysis. (B) Viable cell numbers were counted under the conditions shown in (A). Points represent the mean and standard deviation of three biological replicates. **p< 0.01, Kruskal–Wallis analysis.


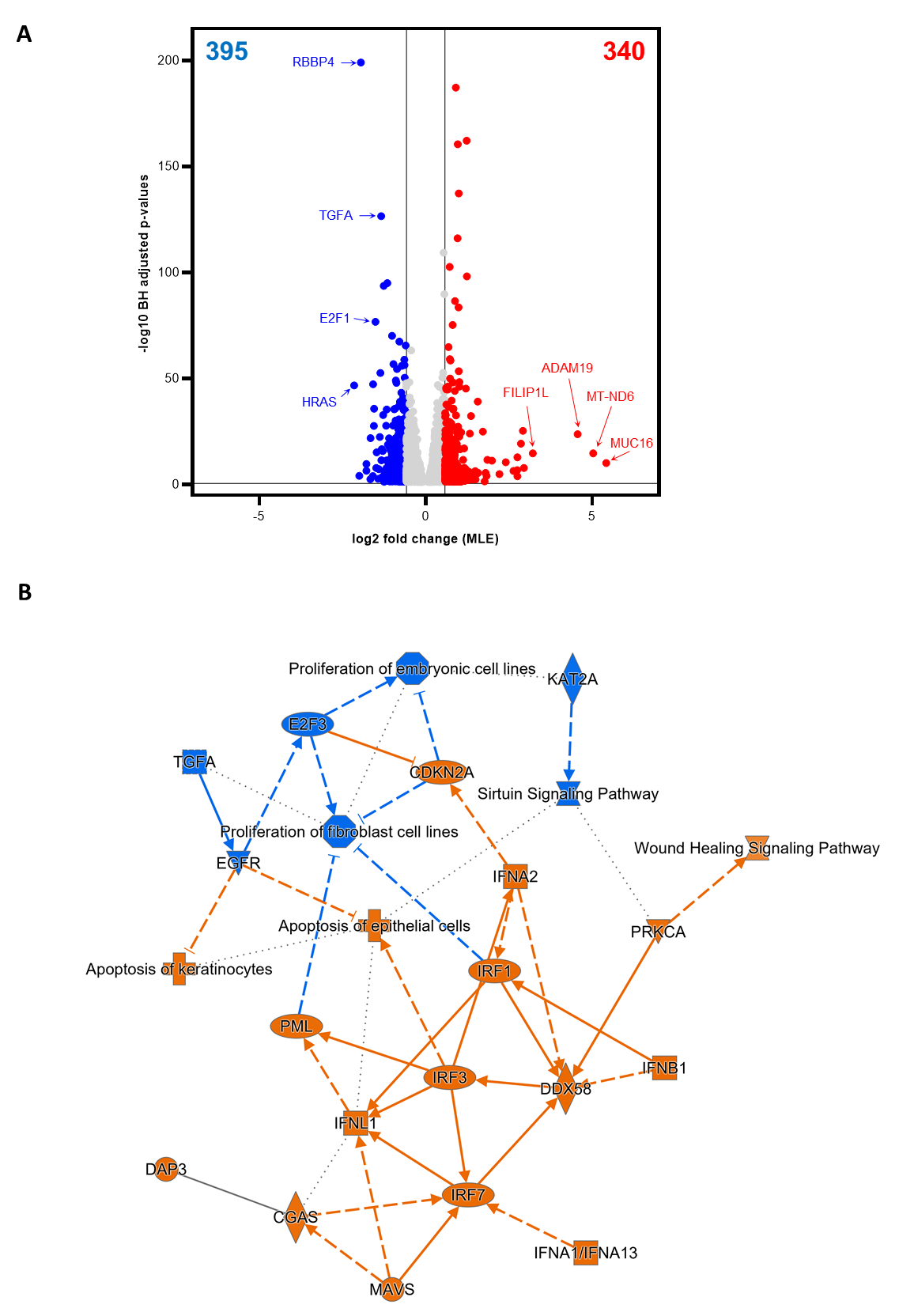


**Supplemental Figure 14. RNA-seq analysis in DAOY cells treated with either si-NC or si-*circ-63706*.**

(A) Volcano plot displaying the differentially expressed genes across referenced with –log10 (P-value) (y-axis) and log2 FC (x-axis). Fold change (FC) >2 and P-value <0.01 were identified as significantly altered. (B) The most significant molecular network by IPA analysis.

**
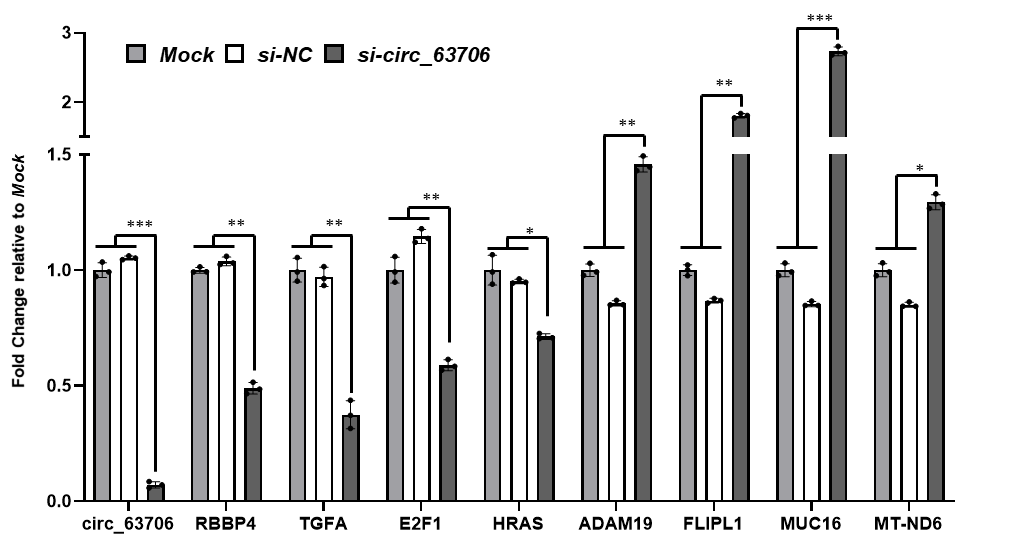
**

**Supplementary Figure 15. qPCR validation of RNA-sequencing data.**

Expression levels of circ-63706*, RBBP4, TGFA, E2F1, HRAS, ADAM19, FLIPL1, MUC16,* and *MT-ND6* were examined by qPCR in DAOY cells treated with either si-*NC* or si-circ-63706. Relative expression level to mock is indicated in the y-axis (n=3). Error bars indicate s.e.m. ****p*< 0.001, ***p*< 0.01, **p*< 0.05, Kruskal–Wallis analysis.

**
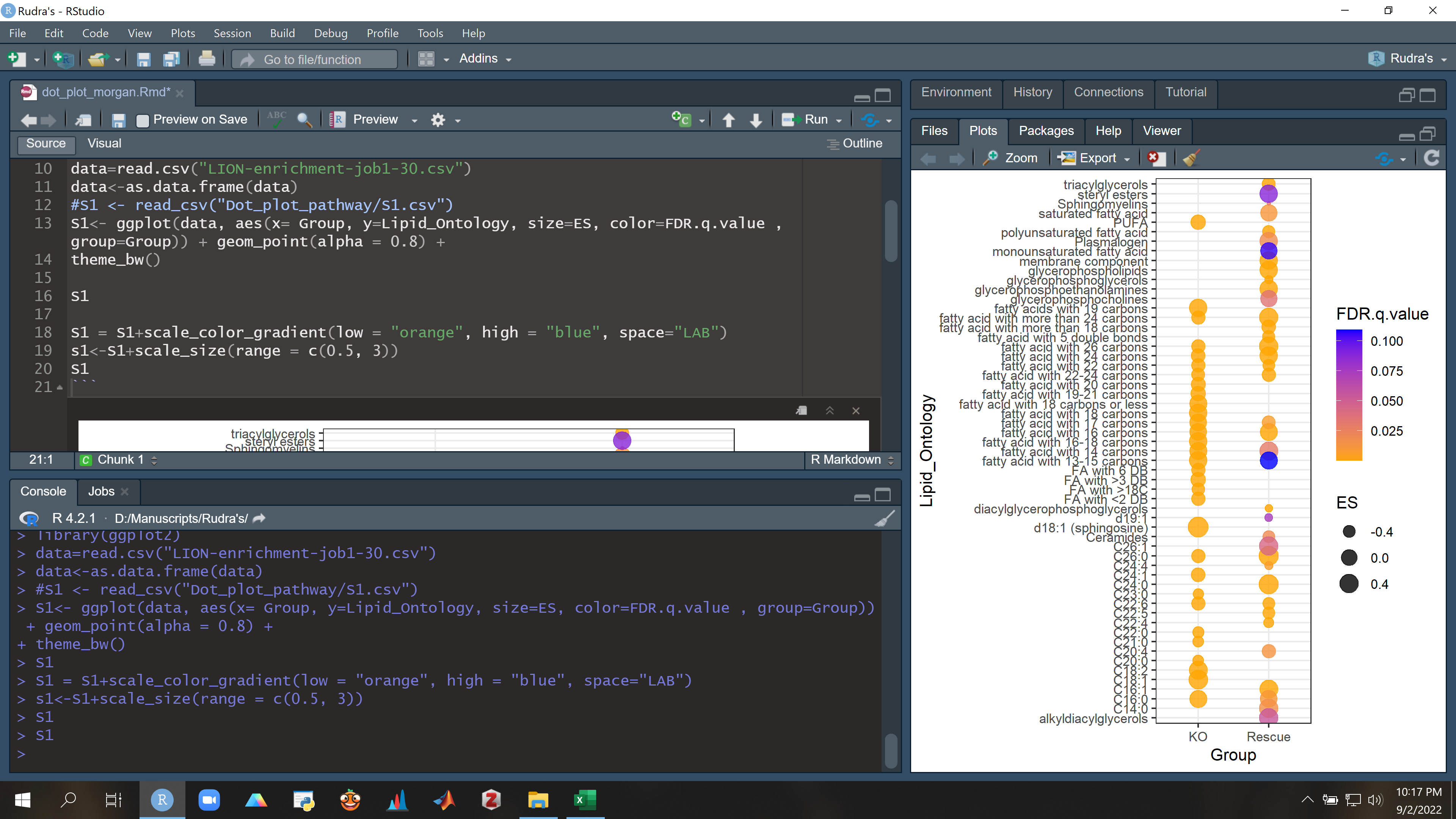
Supplementary Figure 16. Lipid ontology analysis in *circ-63706* expressing MB cells.**

Reprogramming of fatty acid metabolism in *circ-63706* KO and rescued MB cells. The size of the dot indicates the enrichment score (ES), and the color represents significant lipid ontology based on the FDR q-value.
