# Supplementary material for: The oncogenic circular RNA circ_63706 is a potential therapeutic target in sonic hedgehog-subtype childhood medulloblastomas
Source: Acta Neuropathol Commun. 2023 Mar 10;11:38. doi: 10.1186/s40478-023-01521-0 (PMC10007801; doi:10.1186/s40478-023-01521-0)

**Supplementary Tables**

**
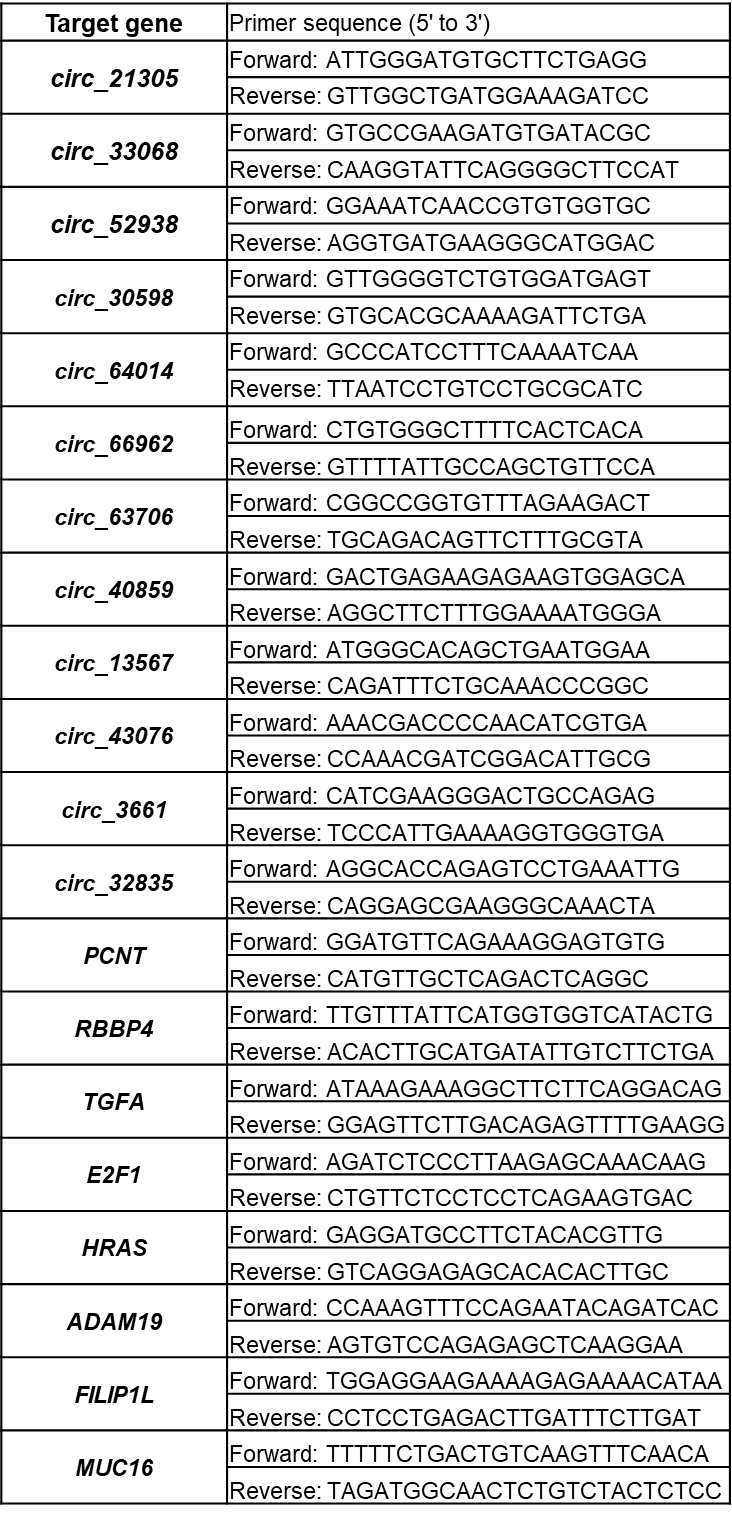
Supplementary Table 1. Primer sequences for qRT-PCR**

**Supplementary Table 2. The 65 medulloblastoma subgroup-specific circRNAs in patient samples**.

| **WNT** | | |  | **SHH** | | |  | **Group3** | | |  | **Group4** | | |
| --- | --- | --- | --- | --- | --- | --- | --- | --- | --- | --- | --- | --- | --- | --- |
| **circRNA** | **logFC** | **adj.P.Val** |  | **circRNA** | **logFC** | **adj.P.Val** |  | **circRNA** | **logFC** | **adj.P.Val** |  | **circRNA** | **logFC** | **adj.P.Val** |
| circ_71350 | 6.55 | 9.31E-32 |  | circ_30598 | 5.62 | 6.29E-23 |  | circ_13567 | 5 | 1.10E-35 |  | circ_21305 | 3.07 | 5.75E-12 |
| circ_51087 | 6.18 | 1.32E-28 |  | circ_64014 | 5.3 | 2.06E-30 |  | circ_40859 | 4.4 | 2.50E-13 |  | circ_72082 | 3.07 | 2.90E-09 |
| circ_55605 | 6.1 | 1.18E-17 |  | circ_45001 | 5.23 | 9.85E-20 |  | circ_43076 | 4.37 | 3.86E-33 |  | circ_56936 | 2.62 | 4.82E-12 |
| circ_78614 | 5.94 | 1.99E-32 |  | circ_4097 | 4.89 | 2.51E-22 |  | circ_32835 | 3.39 | 2.31E-12 |  | circ_29196 | 2.48 | 8.15E-07 |
| circ_39796 | 5.85 | 4.19E-29 |  | circ_66962 | 4.79 | 1.91E-24 |  | circ_30459 | 2.57 | 1.09E-07 |  | circ_74145 | 2.42 | 6.03E-07 |
| circ_42444 | 5.56 | 4.52E-12 |  | circ_9649 | 4.46 | 2.17E-18 |  | circ_60461 | 2.57 | 4.11E-07 |  | circ_77438 | 2.42 | 1.12E-06 |
| circ_50345 | 5.48 | 1.42E-23 |  | circ_28243 | 4.25 | 1.98E-17 |  | circ_62787 | 2.48 | 3.97E-06 |  | circ_33068 | 2.4 | 3.93E-06 |
| circ_68037 | 5.28 | 1.10E-19 |  | circ_63706 | 4.05 | 2.32E-15 |  | circ_7624 | 2.4 | 1.25E-09 |  | circ_52938 | 2.39 | 3.96E-09 |
| circ_62407 | 5.22 | 2.29E-17 |  | circ_1701 | 3.75 | 1.06E-11 |  | circ_68493 | 2.31 | 3.94E-07 |  | circ_63166 | 2.34 | 6.44E-08 |
| circ_76616 | 5.19 | 1.42E-23 |  | circ_11342 | 3.62 | 5.57E-14 |  | circ_28884 | 2.29 | 1.24E-05 |  | circ_61700 | 2.32 | 7.61E-06 |
| circ_44532 | 5.06 | 6.57E-23 |  | circ_38659 | 3.54 | 2.32E-15 |  | circ_3661 | 2.23 | 4.89E-05 |  | circ_71440 | 2.3 | 7.12E-06 |
| circ_66175 | 5 | 1.89E-20 |  | circ_78110 | 3.42 | 3.37E-13 |  | circ_30848 | 2.11 | 1.69E-05 |  | circ_104 | 2.29 | 7.98E-07 |
| circ_42912 | 4.97 | 1.99E-19 |  | circ_17346 | 3.4 | 2.16E-15 |  | circ_77823 | 2.04 | 7.85E-06 |  | circ_65941 | 2.27 | 0.0001307 |
| circ_41600 | 4.94 | 1.66E-19 |  | circ_61423 | 3.34 | 2.09E-12 |  |  |  |  |  | circ_63542 | 2.25 | 5.33E-06 |
| circ_56467 | 4.91 | 4.13E-17 |  | circ_30078 | 3.34 | 3.42E-14 |  |  |  |  |  | circ_39940 | 2.25 | 1.09E-07 |
| circ_74804* | 1.55 | 4.14E-20 |  | circ_74154* | 1.27 | 3.41E-14 |  |  |  |  |  | circ_11865* | 1.26 | 3.13E-13 |
| circ_77990* | 1.59 | 4.55E-22 |  | circ_30078* | 1.39 | 2.74E-18 |  |  |  |  |  | circ_7196* | 3.12 | 4.76E-06 |
|  |  |  |  |  |  |  |  |  |  |  |  | circ_73901* | 1.27 | 5.25E-07 |
| *; CircRNAs from RF Classifier analysis | | | | | | |  |  |  |  |  |  |  |  |

**
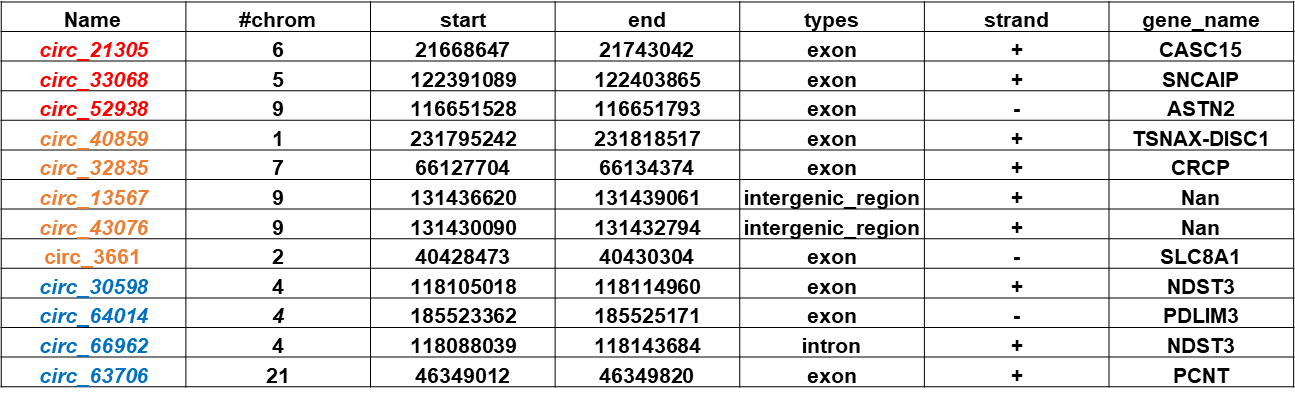
Supplementary Table 3. Top twelve circRNAs for RT-qPCR validation**

**Supplementary Table 4. CT values for circular RNAs in cell line and PDX samples.**

**
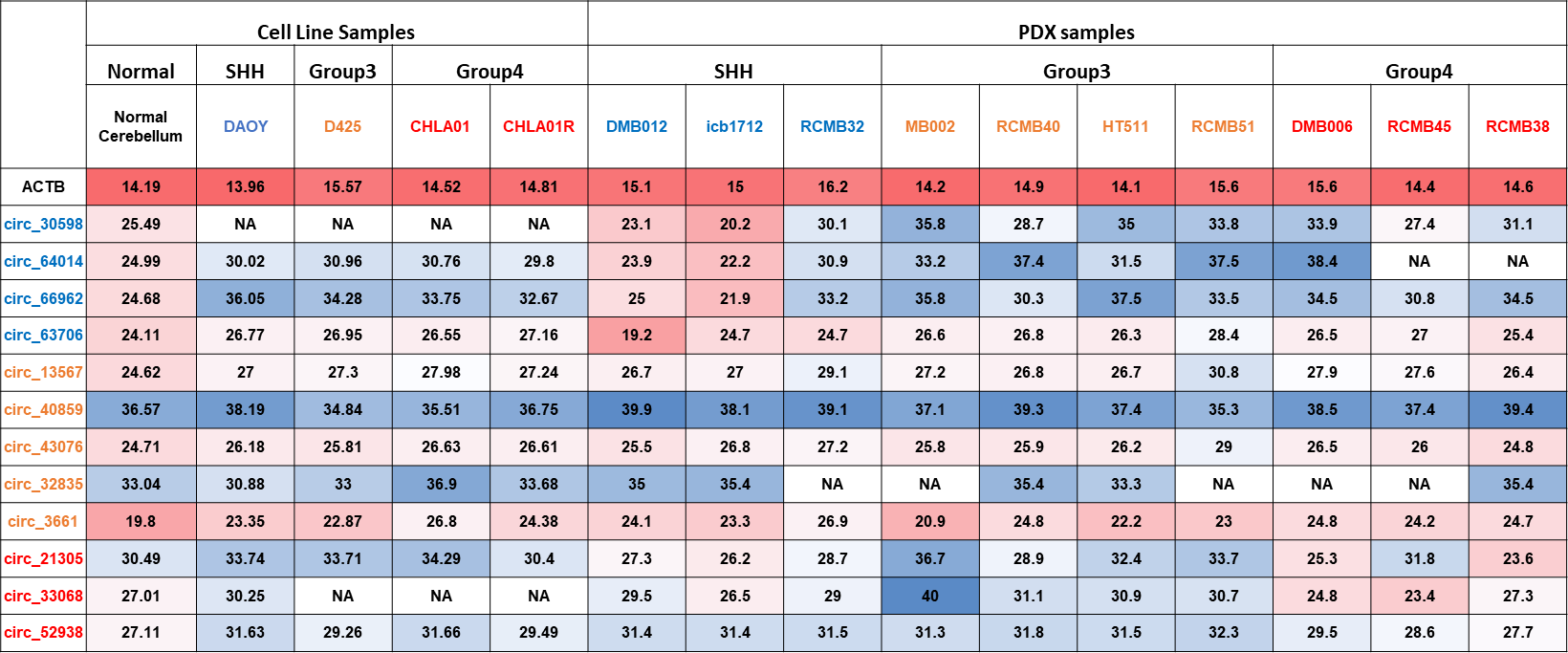
**

**Supplementary Table 5. Coding potential of circRNAs predicted by RNAsamba.**

**
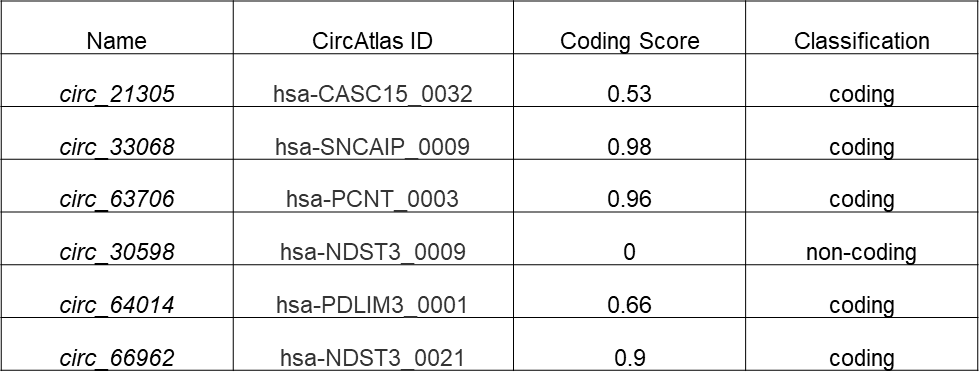
**

**Supplementary Table 6. List of miRNAs that can potentially bind with *circ_63706,* along with their binding energies and the binding sites.**


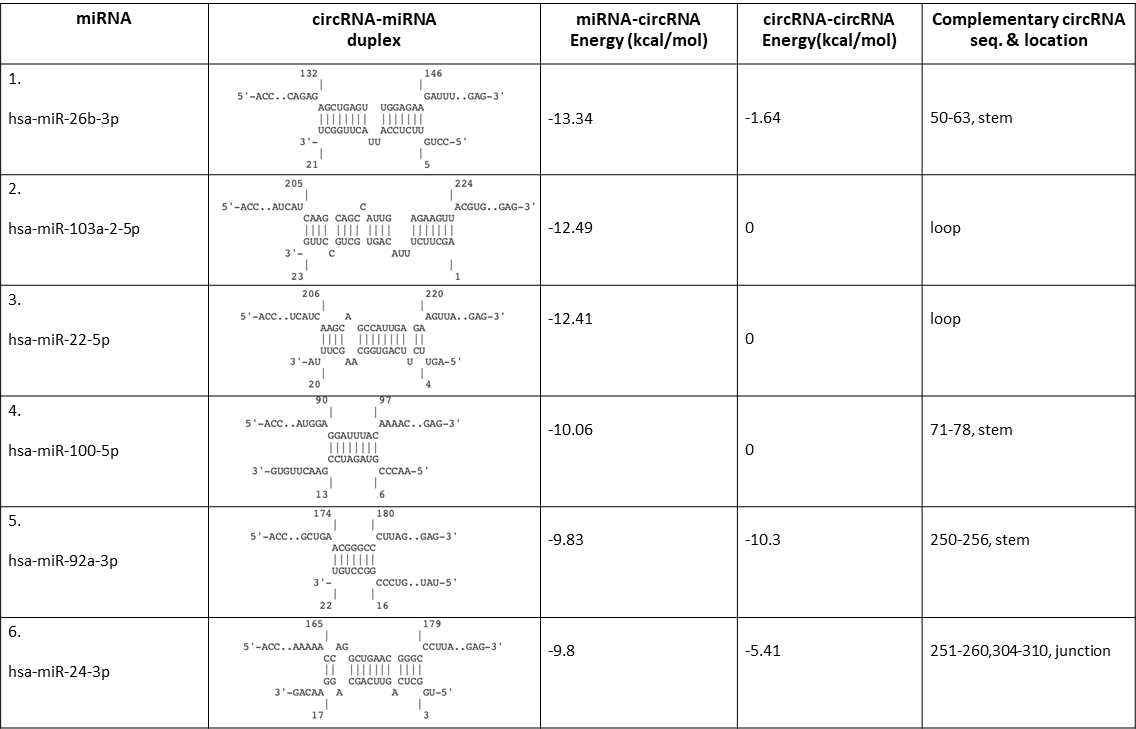

Supplement: Supplementary file 4 — Additional file 4: Supplementary Tables. [file 40478_2023_1521_MOESM4_ESM.docx]
